# Cyclosporine-insensitive mode of cell death after prolonged myocardial ischemia: Evidence for sarcolemmal permeabilization as the pivotal step

**DOI:** 10.1371/journal.pone.0200301

**Published:** 2018-07-05

**Authors:** Katie J. Sciuto, Steven W. Deng, Paul W. Venable, Mark Warren, Junco S. Warren, Alexey V. Zaitsev

**Affiliations:** 1 Nora Eccles Harrison Cardiovascular Research and Training Institute, University of Utah, Salt Lake City, Utah, United States of America; 2 Department of Bioengineering, University of Utah, Salt Lake City, Utah, United States of America; 3 Department of Internal Medicine, School of Medicine, University of Utah, Salt Lake City, Utah, United States of America; Indiana University School of Medicine, UNITED STATES

## Abstract

A prominent theory of cell death in myocardial ischemia/reperfusion (I/R) posits that the primary and pivotal step of irreversible cell injury is the opening of the mitochondrial permeability transition (MPT) pore. However, the predominantly positive evidence of protection against infarct afforded by the MPT inhibitor, Cyclosporine A (CsA), in experimental studies is in stark contrast with the overall lack of benefit found in clinical trials of CsA. One reason for the discrepancy might be the fact that relatively short experimental ischemic episodes (<1 hour) do not represent clinically-realistic durations, usually exceeding one hour. Here we tested the hypothesis that MPT is not the primary event of cell death after prolonged (60–80 min) episodes of global ischemia. We used confocal microcopy in Langendorff-perfused rabbit hearts treated with the electromechanical uncoupler, 2,3-Butanedione monoxime (BDM, 20 mM) to allow tracking of MPT and sarcolemmal permeabilization (SP) in individual ventricular myocytes. The time of the steepest drop in fluorescence of mitochondrial membrane potential (ΔΨ_m_)-sensitive dye, TMRM, was used as the time of MPT (T_MPT_). The time of 20% uptake of the normally cell-impermeable dye, YO-PRO1, was used as the time of SP (T_SP_). We found that during reperfusion MPT and SP were tightly coupled, with MPT trending slightly ahead of SP (T_SP_-T_MPT_ = 0.76±1.31 min; p = 0.07). These coupled MPT/SP events occurred in discrete myocytes without crossing cell boundaries. CsA (0.2 μM) did not reduce the infarct size, but separated SP and MPT events, such that detectable SP was significantly ahead of MPT (T_SP_ -T_MPT_ = -1.75±1.28 min, p = 0.006). Mild permeabilization of cells with digitonin (2.5–20 μM) caused coupled MPT/SP events which occurred in discrete myocytes similar to those observed in Control and CsA groups. In contrast, deliberate induction of MPT by titration with H_2_O_2_ (200–800 μM), caused propagating waves of MPT which crossed cell boundaries and were uncoupled from SP. Taken together, these findings suggest that after prolonged episodes of ischemia, SP is the primary step in myocyte death, of which MPT is an immediate and unavoidable consequence.

## Introduction

Ischemic insult to the myocardium is a major cause of acute or delayed cardiac dysfunction. Reperfusion in a timely fashion is the most effective way to limit infarct size. However, reperfusion itself prompts further myocardial injury. This phenomenon, termed myocardial ischemia-reperfusion (I/R) injury, has been a topic of intensive research for several decades, motivated by strong experimental and (still limited) clinical evidence that pharmacological interventions upon reperfusion, aimed at specific mechanisms in the pathophysiological cascade leading to cell death, can significantly limit the infarct size (see for review [1]).

However, the basic question which remains unresolved is, what is the primary, irreversible step in the death pathway during I/R injury? Older theories postulated that the primary event is the loss of sarcolemmal integrity (we shall use term sarcolemmal permeabilization or SP), presumably a result of sarcolemmal rupture due to hypercontracture, secondary to cellular calcium (Ca^2+^) overload [2] and/or cell swelling [3] upon reperfusion. Electron microscopy evidence suggested that SP is an early event in reperfusion [4].

Crompton and colleagues could be the first to suggest that the process of mitochondrial permeability transition (MPT) due to the opening of the MPT pore is a potential factor of acute myocardial I/R injury [5]. The MPT pore is a non-selective large channel in the inner mitochondrial membrane (with still disputed molecular composition [6, 7]). MPT pore opening is triggered by a combination of increased mitochondrial [Ca^2+^], reactive oxygen species (ROS), and inorganic phosphate [5, 8]. Opening of the MPT pore causes collapse of the mitochondrial membrane potential (ΔΨ_m_) and uncoupling of oxidative phosphorylation, leading to cellular ATP depletion. In addition, it causes mitochondrial matrix swelling and rupture of the outer mitochondrial membrane, resulting in the release of pro-apoptotic factors such as cytochrome c into the cytosol, thereby initiating apoptotic cell death. Mitochondrial peptidyl prolyl cis-trans isomerase, cyclophilin D, sensitizes the MPT pore to Ca^2+^ and confers sensitivity to cyclosporine A (CsA). Thus, CsA increases resistance of MPT to Ca^2+^ [7].

The demonstration that CsA or genetic ablation of cyclophilin D protected ventricular myocytes from cell death in the wake of an ischemic episode [9–11] radically changed the conceptual landscape of myocardial I/R injury. Following those studies, it has been the predominant view that MPT pore opening is the pivotal event in the myocyte death pathway, mainly supported by additional demonstrations of cardioprotection afforded by CsA (for review see [1]). In this prevailing concept, SP is downstream of MPT, but the route from MPT to SP remains obscure [12]. One speculation is that MPT causes critical depletion of ATP and an increase in [Ca^2+^]_i_, leading to irreversible contracture and SP [13]. However, this conjecture has never been demonstrated experimentally. Moreover, a recent study suggested that the hypercontracture-mediated SP may be the primary mechanism of cell injury, at least after relatively brief periods of ischemia [14]. Other studies demonstrated an important role of membrane-stabilizing mechanisms for myocyte survival in I/R injury [15] [16, 17]. It should also be noted that the cardioprotective effect of CsA was not achieved in some experimental models of I/R injury [18–20], and the latest clinical trials failed to demonstrate benefits of CsA in patients undergoing percutaneous coronary intervention (PCI) after myocardial infarction [21, 22]. However, interpretations of the CsA test are complicated by the fact that CsA can only increase the resistance of mitochondria to Ca^2+^, but does not block the MPT pore. Therefore, the lack of CsA effect does not preclude MPT from being involved [7]. For example, a critical increase in sarcolemmal permeability could expose mitochondria to [Ca^2+^] levels high enough to trigger MPT *despite* the presence of CsA.

The purpose of this study was to investigate the temporal, and possible cause-effect relationship, between MPT and SP during reperfusion after prolonged (>60 min) periods of ischemia. This was accomplished by direct visualization of the critical events through time in individual syncytial myocytes using live, confocal microscopy in isolated hearts [16, 23] [24]. We used the cellular loss of mitochondrial membrane potential (ΔΨ_m_)-sensitive dye, TMRM, as the evidence of MPT, and the uptake of cell-impermeable dye, YO-PRO1, as the indicator of SP. We found that under control conditions the putative events of MPT and SP are virtually simultaneous (or overlapping) within the limitations of the method. CsA failed to reduce the infarct size but separated the SP and MPT events such that SP occurred unequivocally ahead of MPT. Extreme cell shortening, consistent with the notion of “hypercontracture” studied in isolated myocytes [2], was rarely observed in whole heart and did not predict the double event of SP/MPT. These findings identify a novel phenomenon of CsA-insensitive abrupt SP during reperfusion after prolonged ischemia. These data are compatible with the idea that after prolonged episodes of ischemia, the SP event represents the irreversible step in myocyte death, in turn triggering MPT.

## Materials and methods

### Ethical approval

The study conformed to the National Institute of Health *Guide for the Care and Use of Laboratory Animals* (8th Edition, 2011) and was approved by the Institutional Animal Care and Use Committee of the University of Utah (Protocol number 14–09009).

### Langendorff–perfused rabbit hearts

New Zealand White Rabbits of either gender (2.5 ± 0.5 kg) were obtained from Western Oregon Rabbitry. Animals were euthanized via intravenous injection of sodium pentobarbital (130 mg/kg) with heparin (10,000 USP units) and both pedal and corneal reflexes were monitored until cessation. Hearts were then quickly excised, cannulated, and Langendorff-perfused with Tyrode solution (130 mM NaCl, 24 mM NaHCO_3_, 1.2 NaH_2_PO_4_, 1.0 mM MgCl_2_, 5.6 mM glucose, 4.0 mM KCl, 1.8 mM CaCl_2_, and 0.1 g/L albumin, pH 7.4) and placed in a custom confocal imaging chamber with a coverslip secured to the bottom. A temperature probe was inserted through the pulmonary artery into the cavity of the right ventricle, and a second probe was set between the posterior surface and the coverslip, close to the imaging area. Flow was held constant (30 mL/min) and temperature was maintained at 37±0.5°C. A bipolar recording electrode was positioned at the border of the left ventricular (LV) posterior imaging area, as well as near the Bachmann bundle of the atria, to monitor both local ventricular and atrial electrical activity throughout the experiment. The global ECG was recorded through silver wires fixed to the bottom of the imaging chamber, located on either side of the heart. Two pairs of pacing electrodes were placed in the LV anterior apex and the intraventricular septum. The pacing current was set at 3x the pre-ischemic end-diastolic excitation threshold for pacing at a constant cycle length of 300 ms during the episode of ischemia.

### Fluorescent dyes and the ischemia-reperfusion protocol

After a stabilization period of 30 minutes, the electromechanical uncoupler butandeone monoxime (BDM, 20 mM) was added to the perfusate to eliminate motion artifacts during confocal imaging, with the exception of beating heart experiments in which no uncoupler was used. Hearts were stained with mitochondrial inner membrane potential (ΔΨ_m_)-sensitive dye, tetramethylrhodamine methyl ester perchlorate (TMRM, 450 nM, excitation/emission 552/578), for 30 minutes, followed by a washout period of 20–30 minutes before the onset of ischemia. The hearts were also stained with YO-PRO1 iodide (333 nM, excitation/emission 491/509). YO-PRO1 was present in the perfusate for 10–15 minutes prior to ischemia and for 2–3 hours during reperfusion. During no-flow ischemia YO-PRO1 remained in the lumen of vessels and in the extracellular space. YO-PRO1 is a normally cell-impermeable nucleic acid stain, entering the cell and binding to nucleic acids when the sarcolemma develops pores large enough to accommodate the size of the YO-PRO1 molecule (Stokes radius ~0.5 nm [25]). YO-PRO1 and similar nucleic acid stains generate extremely low background fluorescence in the extracellular space. However, upon entering permeable cells and binding to nucleic acids, their fluorescence increases over 100-fold, creating the largest signal in the nuclei while also “flooding” the entire cell volume due to binding cytosolic RNA. This tremendous change in fluorescence, along with the small size of the YO-PRO1 molecule, renders this indicator ideal for efficient detection of SP.

Global ischemia was initiated by stopping aortic perfusion and ventricular pacing was commenced at the cycle length of 300 ms. To maintain temperature during the ischemic period (60–80 min), heaters attached to the imaging chamber walls were employed and hearts were superfused with warm (37°C) Tyrode solution gassed with 95%N_2_/5%CO_2_ to remove oxygen. Upon reperfusion, hearts were re-stained with TMRM for the first 10 minutes to compensate for washout of TMRM released to the extracellular space during ischemia due to a combination of mitochondrial and sarcolemmal depolarization. After 3 hours of reperfusion, some hearts were sliced transversally and stained with 2,3,5-triphenyltetrazolium chloride (TTC) to observe the resulting tissue viability and infarct.

### TTC staining quantification

Following 10 minutes of TTC staining, heart slices were scanned (HP Scanjet 5550c) to a high-resolution image (2400 dpi). Each slice was isolated to form a separate color image, which was then split into the monochrome RGB components. The green channel image is known to provide the highest contrast between infarcted and healthy tissue regions [26], and thus was used in the remaining steps for quantification. The LV was segmented and all remaining pixels were masked out. A histogram of LV pixel intensity was generated, yielding a continuous distribution of intensity corresponding to all shades of color from pure white to cherry red. After careful visual inspection of several hearts, two thresholds were set at intensity values of 90 and 125, separating red, pink, and white regions. Although the exact choice of the thresholds was based on visual impression, they were applied uniformly to all TTC stained images. Pixel counts for values below 90, between 90 and 125, and above 125 were summed to denote viable (healthy), intermediate, and severely infarcted tissue, respectively. The summed pixel counts were divided by the full number of LV pixels to determine the percentage of total tissue for each region (see S1 Fig).

### Experimental groups

All experiments were performed in the presence of BDM, with the exception of the *No-BDM* group (n = 3). Hearts treated with BDM alone (n = 7) served as the main *Control* group. In the *CsA* group (n = 9), CsA (0.2 μM) was present in the perfusate starting from 10 min before ischemia through a minimum of 2 hours of reperfusion. In order to explore the effect of oxidative stress on MPT and SP, in 4 experiments H_2_O_2_ was perfused in increasing concentrations of 200, 400, and 800 μM, until a visible effect in terms of TMRM fluorescence (F_TMRM_) decrease was achieved (*H*_*2*_*0*_*2*_ group). Each concentration was applied for 20 minutes with periods of 20 minutes in between. In 4 experiments we applied the cell-permeabilizing agent digitonin in increasing concentrations of 1, 5, 10, and 20 μM (20 mins each), until the earliest signs of permeabilization (YO-PRO1 uptake) were clearly visible (*Digi* group) and estimated the time interval between YO-PRO1 uptake and TMRM loss. In one additional experiment, we applied the protonophore and mitochondrial uncoupler FCCP. FCCP was applied in increasing concentrations (1, 5, 10, and 20 μM) for 15-minute periods each, with the last step lasting for half an hour.

### Confocal imaging of fluorescent signals

Confocal imaging was performed essentially as described in our previous publication [27], and only modifications will be described in detail. Briefly, the hearts were positioned in an imaging chamber mounted on the stage of an inverted Zeiss LSM 510 confocal microscope, and the posterior LV surface was imaged through a glass coverslip glued to the bottom of the chamber. All images used for data analysis were acquired using a 20x objective lens. A 10x lens was used occasionally to select areas for imaging and to exclude bleaching/phototoxicity in intensively imaged areas. In this study, we used both the conventional (slow) scan mode and the fast scan mode (using Zeiss LSM 5 Live duo module). In the fast scan mode, pixel dwell time was 23.74 μs and the full-frame scan time for each channel was 33.33 ms. Images (12-bit) were acquired with 1024 x 1024-pixel resolution, 335.5 x 335.5 μm field of view, and a pixel size of 0.33 μm. In the conventional scanning mode, the full-frame scan time was 3.95 seconds for a single channel and pixel dwell time was 0.80 μs. Images (12-bit) were acquired with 1024 x 1024-pixel resolution, 450 x 450 μm field of view, and a pixel size of 0.44 μm. In both scan modes, 2D and 3D scans (z-stacks) were utilized. Z-stacks (slice thickness and inter-slice interval, 2.70 μm; 6–10 slices per stack) were acquired with at least 3-minute intervals between acquisitions, while 2D images of F_TMRM_ and F_YO-PRO1_ were taken every 15–60 seconds (1–4 frames per minute). Imaging at these rates avoided laser bleaching that was apparent when more frequent rates were used.

### Analysis of F_TMRM_ and F_YO-PRO1_ during ischemia/reperfusion

Consistent with previous reports [23, 24] we used an abrupt loss of F_TMRM_ occurring in discrete myocytes as the sign of MPT, and tracked the cell-averaged value of F_TMRM_ to determine MPT timing during reperfusion [23, 24]. Because cellular F_TMRM_ loss occurred as an avalanche-like process with sigmoidal dynamics, we used the point of the steepest slope of F_TMRM_ drop as an estimate of the time of MPT (T_MPT_). In contrast, cellular uptake of YO-PRO1, signifying SP, was usually gradual and continuous without a clear point of fastest transition. As an estimate of the time of SP we used the point at which the average F_YO-PRO1_ increased to 20% of the total range of F_YO-PRO1_ during the period of observation. These time-point thresholds were vital to objectively determine when MPT and SP occurred, while eliminating conceivable false detection elements due to potential motion or drift in the z-plane.

The observation of cell transitions during reperfusion was a random and serendipitous event, because (a) it occurred in discrete myocytes in an unpredictable manner, and (b) the field of view and the focal plane could shift at any instance of time due to slow changes in heart shape occurring during reperfusion. Among all cells in which the abrupt loss of F_TMRM_ during reperfusion was captured, we selected those which were imaged for at least 3 min before the start of the fast F_TMRM_ drop and at least 5 min after this event. Also, cases in which F_TMRM_ decrease could be attributed to bleaching due to excessive laser exposure were excluded. The bleaching was detected by zooming out (switching from the standard 20x lens to the 10x lens) and comparing F_TMRM_ inside and outside the field of view imaged with the 20x lens (see S2 Fig).

### Statistics

We used one-sample Student t-test to determine whether an interval between two events of interest was significantly different from zero, and Grubbs test to identify significant outliers. Statistical analysis was performed using XLSTAT software by Addinsoft (www.xlstat.com). The data are presented as mean ± standard deviation. Differences with p < 0.05 were considered statistically significant.

## Results

We first analyzed the global dynamics of F_TMRM_ and F_YO-PRO1_ during ischemia and reperfusion. During ischemia, we observed a global loss of ΔΨ_m_ consistent with our previous publication [27], but did not observe SP (see S1 Text, S3 Fig and S1 Movie). During reperfusion, the first event was recovery of ΔΨ_m_. After that, some cells exhibited undisputable ΔΨ_m_ loss and SP. We focused further analysis on the timing of critical cellular transitions in individual ventricular myocytes during reperfusion.

### Analysis of F_TMRM_ and F_YO-PRO1_ dynamics in individual cells during reperfusion

In a total of 7 *Control* experiments we captured 26 cellular transition events in which individual myocytes lost F_TMRM_ and gained F_YO-PRO1_ during reperfusion. This was observed in a wide range of delays with respect to the moment of reperfusion (from 9 to 131 min). Our ability to catch events in the first few minutes of reperfusion was limited due to excessive drifts in the field of view caused by heart expansion. However, within the range when events were observable, there was no obvious difference between events occurring early or late in reperfusion. From the total of 26 cells exhibiting critical transitions, 13 cells were selected according to criteria indicated in Methods.

**Fig 1:**
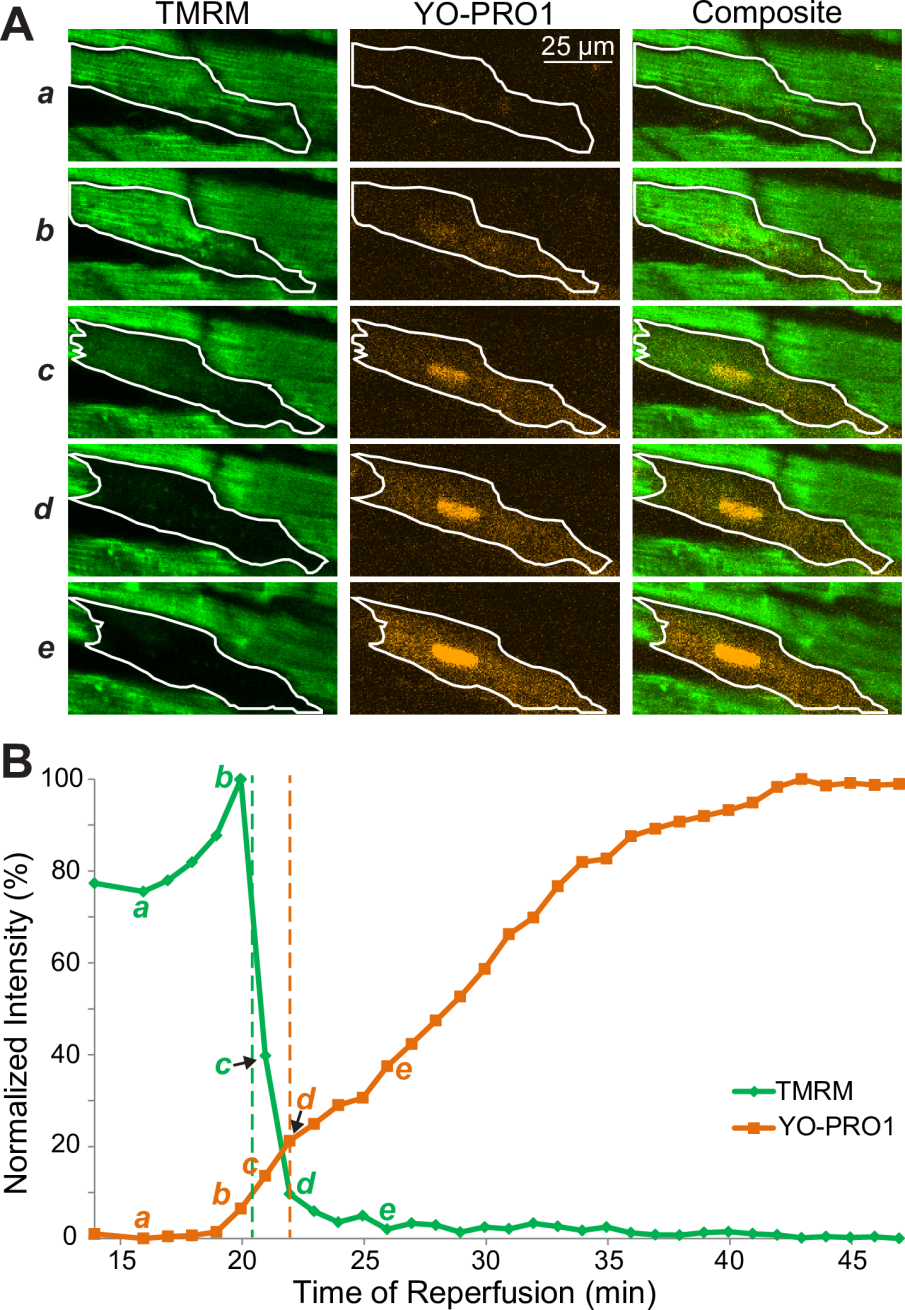
Detailed spatiotemporal analysis of F_TMRM_ loss and F_YO-PRO1_ gain during reperfusion in a representative myocyte from a control heart. **A**, each row shows F_TMRM_ (green), F_YO-PRO1_ (orange) and the merged image of the cell undergoing the critical transition (outlined with white) for different time points (*a* to *e*) indicated in **B**. **B**, the cell-averaged F_TMRM_ (green) and F_YO-PRO1_ (orange) as the function of time shown as 0–100% of the dynamic range of each signal. The vertical green and orange dashed lines indicate T_MPT_ and T_SP_, respectively. Note the spatiotemporal overlap between the processes of F_TMRM_ loss (indicator of MPT) and F_YO-PRO1_ gain (indicator of SP), such that at time point *b* there is a clear uptake of YO-PRO1 while the majority of the cell interior still shows polarized mitochondria.

Fig 1 shows a representative example of the analysis of F_TMRM_ and F_YO-PRO1_ dynamics in a single myocyte (the full sequence of frames is shown in S2 Movie). In Panel A, each row shows F_TMRM_ (green), F_YO-PRO1_ (orange) and the merged image for a small region of interest depicting the cell undergoing the critical transition (outlined with white). Rows from top to bottom represent different time points (*a* to *e*) indicated in Panel B. Panel B shows the cell-averaged F_TMRM_ (green) and F_YO-PRO1_ (orange) as the function of time. At time point *a* (16 min of reperfusion), the cell of interest portrays well polarized mitochondria with a preserved grid-like pattern of TMRM staining, and no evident YO-PRO1 presence in the cell. At time point *b* (20 min of reperfusion), noticeable events begin to occur at the same time. First, there is a decrease in F_TMRM_ level in the right end of the cell simultaneous with the apparent loss of the grid-like pattern (smoothening) of F_TMRM_ in the rest of cell. Second, there is a faint but appreciable increase in F_YO-PRO1_ in the right and central portion of the cell, but probably not yet in the left end of the cell. A minute later (time point *c*), there is a clear overlap between F_TMRM_ and F_YO-PRO1_ in the cell, which is well visible in the merged image. At this point YO-PRO1 reaches the nucleus, producing a large increase in F_YO-PRO1_. The mid-point between *b* and *c* is used as the objective estimate of T_MPT_ (vertical green line). Finally, at time point *d* and beyond, F_TMRM_ becomes indistinguishable from background, whereas F_YO-PRO1_ reaches the 20% level (T_SP_; vertical orange line) and continues to rise. Thus, using a rather conservative definition for T_SP_, in this case MPT evidently occurred slightly ahead of SP. However, subjectively these events appeared to be simultaneous.

The cell-averaged F_TMRM_ and F_YO-PRO1_ dynamics in all 13 cells are shown is S4 Fig. One cell (cell #5 in S4 Fig) was excluded as a significant outlier according to Grubbs test. In the remaining cells T_MPT_ trended to be slightly ahead of T_SP_, however, this was not significant (T_SP_-T_MPT_ = 0.76±1.31 min; p = 0.07). Overall, in the majority of analyzed cells MPT and SP occurred within less than 1 minute of each other in either order, and the events clearly appear to overlap in time.

Since BDM might affect the pattern of cell injury in I/R [14, 28], we attempted to image cell transitions in beating hearts *(No_BDM* group) using the fast frame mode (frame interval 33 ms, see Methods). However, these attempts proved to be impractical because of the extremely fast bleaching and signs of phototoxicity observed under these conditions, evidently due to much larger cumulative laser exposure than when using conventional scan mode. However, the fast frame mode enabled us to obtain sharp snapshot images from beating hearts due to short frame scan time. These images demonstrated mutual exclusion between cells showing F_TMRM_ or F_YO-PRO1_ and the lack of cells devoid of both signals (no “black cells”, see S5 Fig). These findings suggest that in beating hearts, myocytes gained F_YO-PRO1_ as soon as F_TMRM_ was lost, which is consistent with the coupled F_TMRM_ loss and F_YO-PRO1_ gain observed in the *Control* hearts treated with BDM.

Overall, these outcomes indicate that MPT and SP are tightly coupled, so that whichever event occurs first, the other event follows right away. We surmised that if MPT is the primary event, then the administration of MPT pore inhibitor CsA should impede both MPT and SP, or at least affect the temporal relationship between the two events.

### Cyclosporine A separates SP and MPT events but does not reduce infarct size after prolonged ischemia

**Fig 2:**
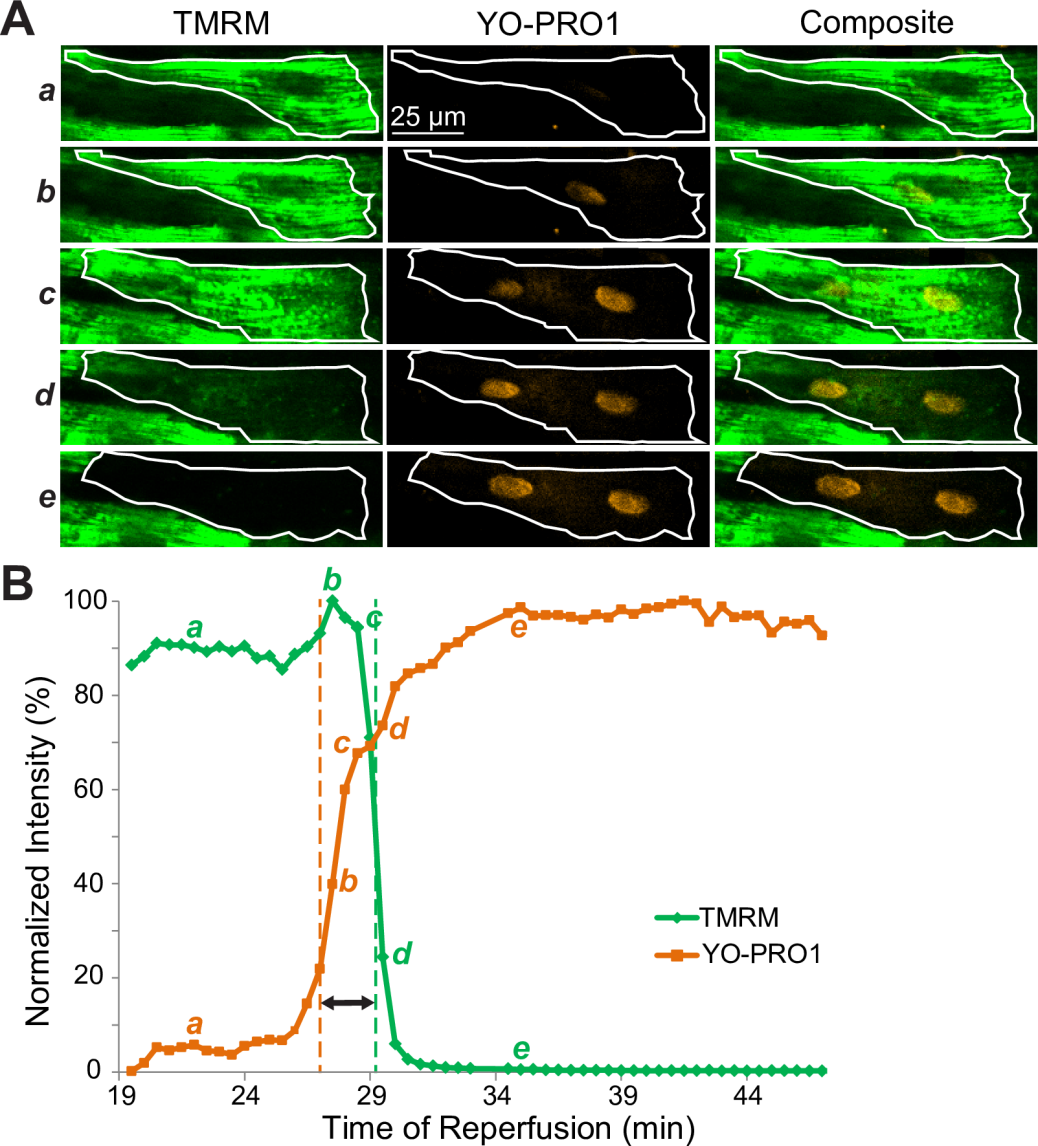
Detailed spatiotemporal analysis of F_TMRM_ loss and F_YO-PRO1_ gain during reperfusion in a myocyte from a heart treated with CsA (0.2 μM). Notations are the same as in Fig 1. Note that compared to a myocyte from a control heart shown in Fig 1, the earliest detectable F_YO-PRO1_ gain (indicator of SP), which is observed at the time point *b*, clearly occurs before the processes of F_TMRM_ loss (indicator of MPT).

Fig 2 shows a representative example of the analysis of F_TMRM_ and F_YO-PRO1_ dynamics in a single myocyte in a heart from the *CsA* group. (The full sequence of frames is shown in S3 Movie). The layout is the same as in Fig 1.

One can see that overall, the sequence of events is similar to that in *Control* (Fig 1). However, the overlap between F_TMRM_ loss and F_YO-PRO1_ gain is more prominent. YO-PRO1 reaches the nucleus at time point b, while F_TMRM_ remains largely unchanged. Unequivocally, detectable SP occurs before detectable MPT. Cell-averaged signals in Fig 2B show that the formally defined SP event (orange line) occurs about 2 minutes before MPT (green line). This principal finding was confirmed in a total of 8/9 analyzed individual cardiomyocytes, with T_SP_-T_MPT_ being significantly negative (SP ahead of MPT; -1.75±1.28 min, p = 0.006). One cell showing an unusually large gap between SP and MPT (T_SP_-T_MPT_ = -13.25 min) was excluded as an outlier according to Grubbs test.

**Fig 3:**
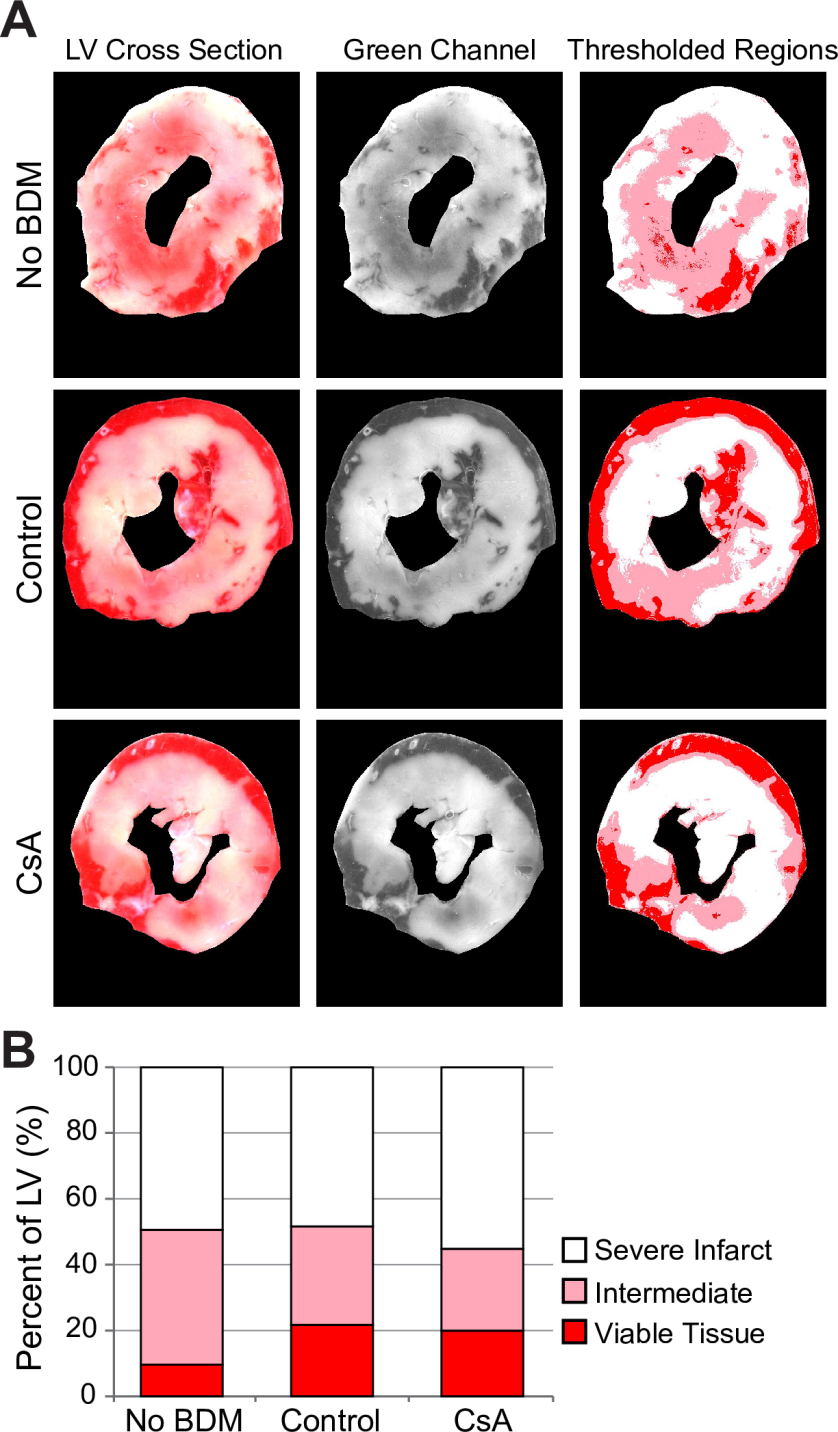
Assessment of infarct size using TTC staining. **A**, *left to right*, examples of original scanned images of TTC staining, the respective green channel signal, and the thresholded regions, respectively (see detailed description of TTC staining procedure in S1 Fig). Rows from top to bottom in **A** represent *No_BDM*, *Control*, and *CsA* groups, respectively. **B**, average percent of severe infarct (white), intermediate infarct (pink) and viable tissue (red) in the three experimental groups. The error bars are omitted for clarity, but none of the regions were significantly different between the groups by 2-way ANOVA. At least in part this is due to the large variation in TTC staining data between hearts in each group (see S6 Fig).

Whereas CsA separated the events of MPT and SP, it did not prevent either of these phenomena in microscopic images. We hypothesized that CsA fails to protect against infarct in our model. We assessed the total infarct size in the left ventricle using TTC staining in *No_BDM*, *Control*, and *CsA* groups. We objectively quantified TTC staining using fixed thresholds in the color distribution dissecting “viable” (red), “severely infarcted” (white) and “intermediate” (pink) areas (see Methods). Fig 3 shows the average distribution between the three levels of injury in the 3 groups. Although on average the *No_BDM* group appeared to have less healthy and more intermediately affected myocardium than the BDM group, there was no statistically significant difference in the percentages of each level of injury between the three groups by ANOVA test. This indicated that variance within groups was larger than the variance between groups. Indeed, in each group there was a large variability in TTC staining distribution between individual hearts as well as individual sections in each heart. Considering individual sections pooled from all hearts, the percentage of severely infarcted area ranged between 11 and 93% in *No_BDM* hearts, between 10 and 93% in *Control* hearts, and between 2 and 98% in *CsA* hearts (see S6 Fig). In each group, however, there was a significant gradient in the percentage of severely infarcted area between the most basal and the most apical sections, with the base consistently showing less infarct than the apex (see S6 Fig).

**Fig 4:**
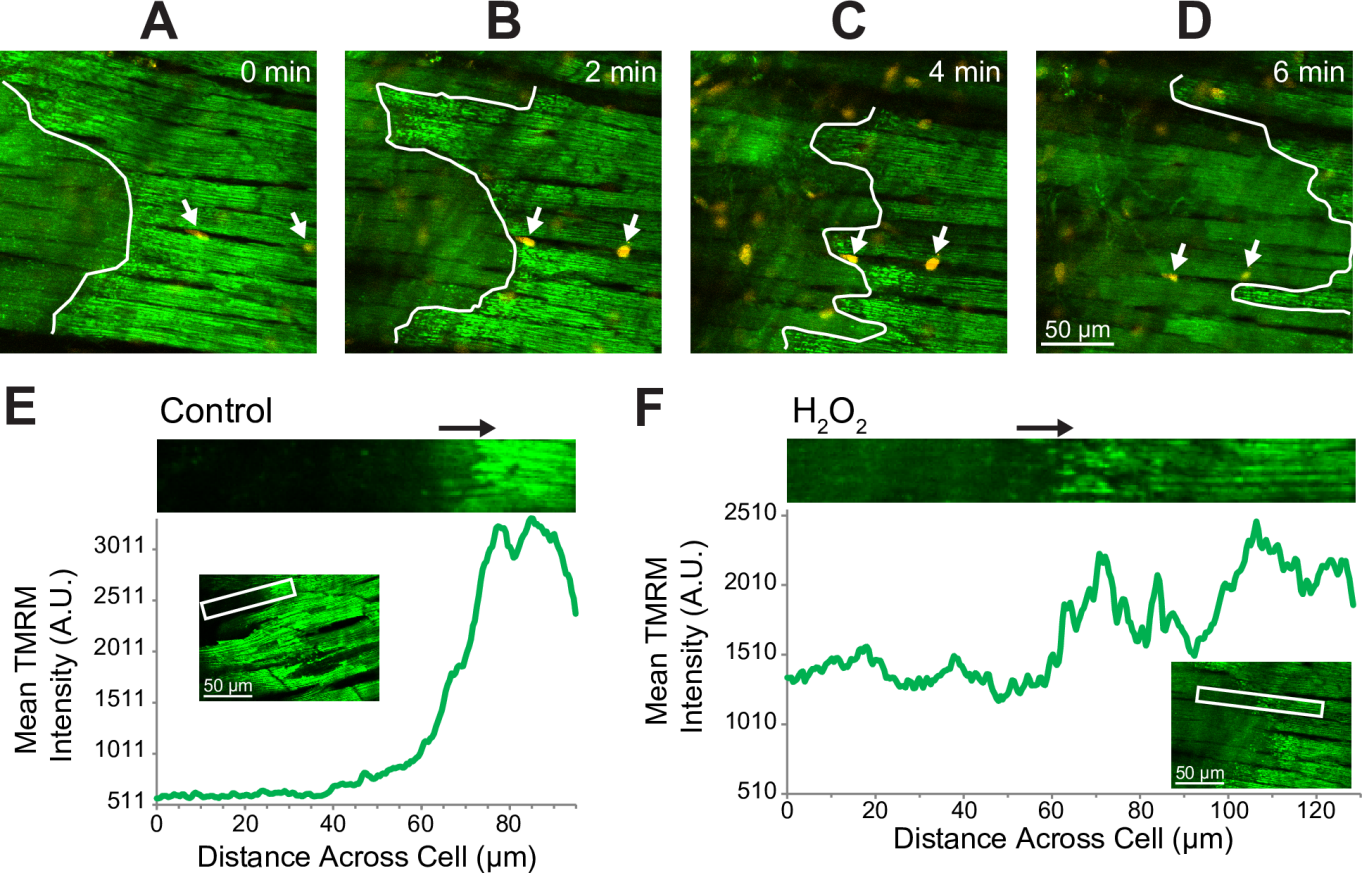
Wave-like propagation of MPT caused by H_2_O_2_. The time is shown according to the onset of observation starting 6 min after the end of H_2_O_2_ application (200 μM). **A** to **D**, consecutive snapshots taken every 2 minutes. Green, fluorescence of TMRM (F_TMRM_); orange, fluorescence of YO-PRO1 (F_YO-PRO1_). The nature of objects brightly stained with YO-PRO1 is unknown, but they may represent dead fibroblasts or other cell types which are usually abundant in the most superficial layers and are visible immediately after YO-PRO1 delivery to the heart. White arrows point to two such bright objects, which were used as fiducial points to track myocytes amid slight changes in the field of view and the focal plane. Note that unlike the naturally occurring MPT/SP events, H_2_O_2_-induced MPT propagated as a wide front (white line), easily crossing cell boundaries, and was not associated with immediate cellular YO-PRO1 uptake. **E** and **F**, Different structure of the propagating front of _FTMRM_ loss during naturally occurring MPT (**E**; same cell as #13 in S4 Fig as well as in S5 Movie) and H_2_O_2_-induced MPT (**F**). In each Panel, F_TMRM_ was computed as the function of the distance along the long axis of the rectangular region of interest (ROI) approximating the selected cell (white rectangle). The magnified image of the ROI is shown at the top of each Panel. For each x-position along the cell, F_TMRM_ was averaged over all pixels in the respective column (across the width) of the rectangle. This yielded an instantaneous profile of the propagating wave (green curve). The minimal value on the y-axis of mean F_TMRM_ represents the minimal value in the field of view (extracellular level, or background). Note that during naturally occurring MPT (**E**) the level of F_TMRM_ in the wake of the MPT front is very close to the background level indicating complete loss of the TMRM in that part of the cell. In contrast, during H_2_O_2_-induced MPT (**F**) the level of F_TMRM_ in the wake of the MPT front remains at about 50% of the dynamic range.

We also checked to which extent a deliberate induction of MPT by H_2_O_2_ [29] would cause SP. We found that H_2_O_2_ caused a principally different phenomenon, a propagating front of ΔΨ_m_ loss which freely crossed cell boundaries and was not followed by SP for at least the next 5 minutes (see Fig 4A–4D and S4 Movie). Even though the naturally occurring MPT also propagated as a wave and usually started at a cell end (see S7 Fig), it was limited to one cell at a time and was overlapping with SP (see Fig 1 and S2 Movie). Also note that in the wake of propagating MPT caused by H_2_O_2_, TMRM was partly retained by the cell, whereas in in the wake of propagating MPT caused by I/R the cellular TMRM signal dropped to a level indistinguishable from background (see Fig 4E and 4F). This again underscores the phenomenological difference between natural and H_2_O_2_ -induced MPT (see more in Discussion). Application of the protonophore, FCCP, caused an expected global dissipation of ΔΨ_m_ but no detectable SP (see S8 Fig).

Summarizing the above, after 80 min of no-flow ischemia, CsA did not afford protection against infarct. CsA did, however, change the mutual dynamics of SP and MPT, separating the two in time such that SP occurred significantly ahead of MPT. This, in combination with the lack of SP occurrence after either MPT induction or ΔΨ_m_ dissipation (H_2_O_2_ and FCCP respectively) suggests that SP is the primary event, and MPT the secondary, in the course of cardiomyocyte injury during reperfusion (see more in Discussion).

### Mild permeabilization of myocytes with digitonin causes loss of TMRM coupled with uptake of YO-PRO1

**Fig 5:**
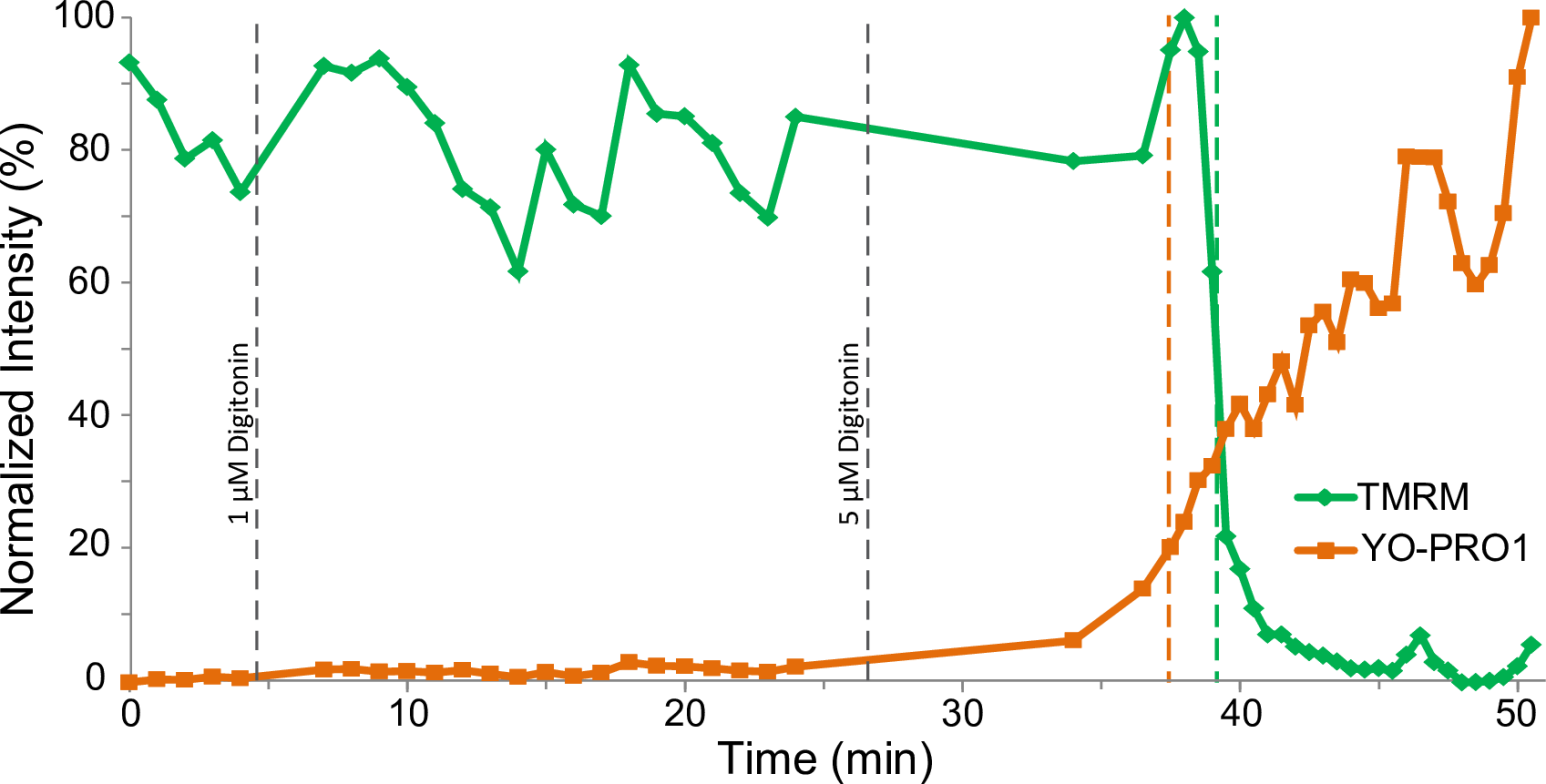
Mutual dynamics of cell-averaged F_TMRM_ loss and F_YO-PRO1_ gain in a cell from a heart subjected to titration by the membrane detergent digitonin (the *Digi* group). The F_TMRM_ loss (indicator of MPT) and F_YO-PRO1_ gain (indicator of SP) overlap in time, reminiscent of cell transitions occurring during reperfusion in the absence (see Fig 1) and the presence (see Fig 2) of CsA.

While the findings presented above speak against the notion that MPT is the cause of SP in this model, we also sought to demonstrate the converse, i.e., that permeabilization of sarcolemma may cause an immediate event of MPT. In the *Digi* group, we titrated hearts with increasing concentrations of the detergent digitonin until the uptake of YO-PRO1 became detectable. Fig 5 shows cell-averaged F_TMRM_ (green) and F_YO-PRO1_ (orange) in a representative example of digitonin treatment. The observed event occurred approximately 11 minutes after addition of 5 μM digitonin into the perfusate. Note the overall similarity of the mutual F_TMRM_ and F_YO-PRO1_ dynamics caused by digitonin to that observed during naturally occurring reperfusion events (see Fig 1).

**Fig 6:**
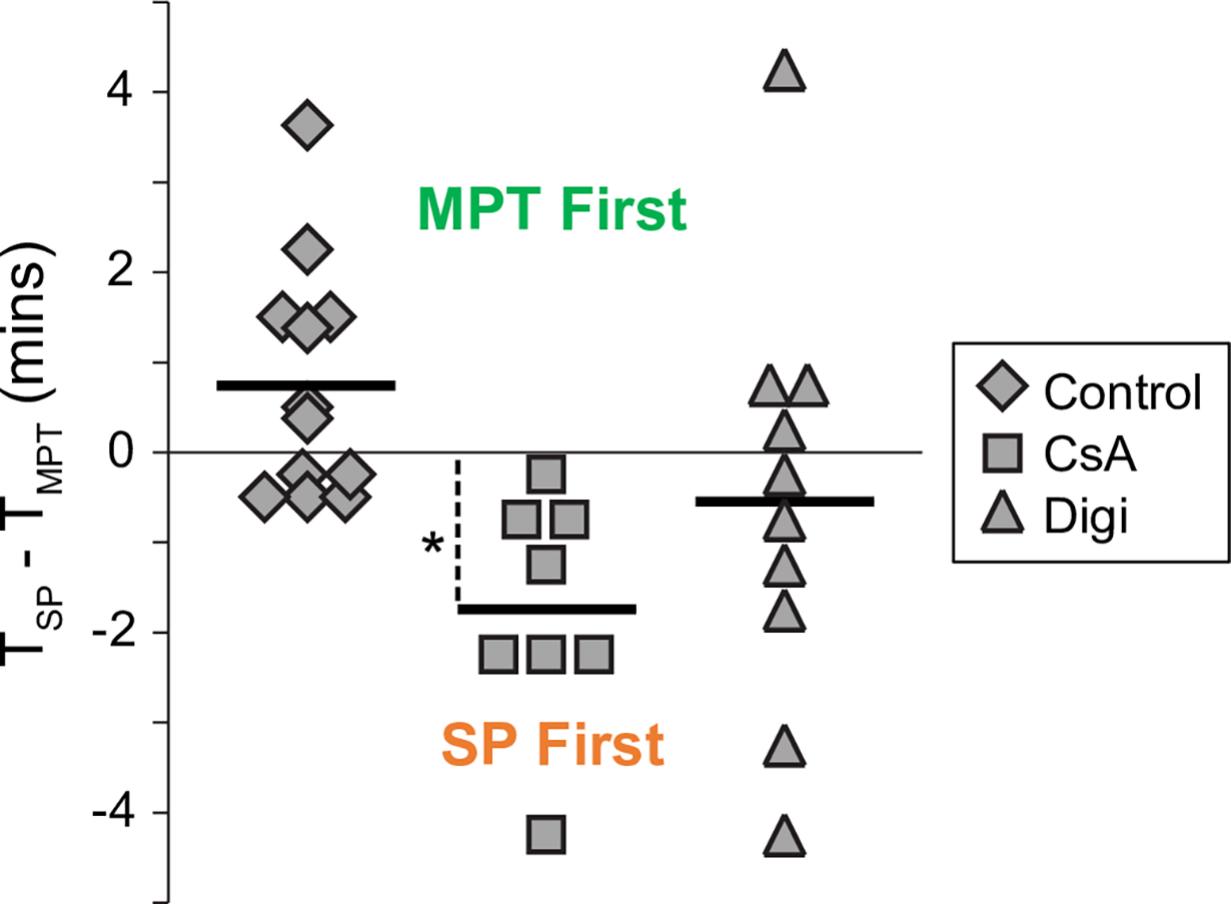
The summary of the time difference between the MPT and SP events (T_SP_-T_MPT_) in individual myocytes from the three experimental groups as indicated. Thick horizontal bars show average values. Note that CsA increases separation between MPT and SP, such that T_SP_-T_MPT_ is significantly negative. *, p < 0.01.

Fig 6 summarizes the T_SP_-T_MPT_ data from individual myocytes from *Control*, *CsA* and *Digi* groups. Negative values signify that SP was detected before MPT. In *Control* the majority of cells exhibited MPT and SP occurring within a minute of each other, and the difference of average T_SP_-T_MPT_ did not reach statistical significance (T_SP_-T_MPT_ = 0.76±1.31 min; p = 0.07). In the presence of CsA, however, all cells exhibited SP ahead of MPT, which was statistically significant (T_SP_ -T_MPT_ = -1.75±1.28 min, p = 0.006). In *Digi* group the distribution was rather broad, but in the majority of analyzed cells SP occurred ahead of MPT similar to *CsA* group. However, due to large dispersion, the average T_SP_-T_MPT_ was not significantly different from zero (-0.55±2.36 min; p = 0.48).

## Discussion

This study addresses the relationship between the events of SP and MPT in isolated perfused rabbit hearts after prolonged episodes of global no-flow ischemia. The most important novel findings of this study are threefold: (1) in control hearts the apparent event of MPT overlaps with the onset of SP; (2) CsA separates SP from MPT (SP occurs first); (3) mild permeabilization of myocytes with digitonin reasonably reproduces coupled MPT/SP events that naturally occur during reperfusion, whereas induction of MPT by H_2_O_2_ does not reproduce these events.

At least three previous reports [16, 23, 24] concluded that mitochondrial permeability transition (MPT) precedes sarcolemmal permeabilization (SP) during reperfusion after myocardial ischemia, thus asserting the unidirectional cause-effect vector from MPT to SP postulated earlier [13]. However, these previous studies did not provide any estimation of the time difference between the alleged events of MPT and SP. The pioneering work by Matsumoto-Ido et al. [23] was the first to describe MPT manifested as the abrupt loss of F_TMRM_ in discrete syncytial myocytes during reperfusion (and our analysis of F_TMRM_ dynamics corroborates theirs), but they did not estimate the temporal relationship between MPT and SP. The authors stated that “cells that underwent ΔΨ_m_ loss eventually experienced cytolysis” but they did not provide support for the “eventuality” notion, since this statement was based on snapshot images indicating mutual exclusivity of TMRM and propidium iodide (PI) staining. PI is a cell permeability indicator similar to YO-PRO1, but with a larger molecular size. The observation of a reciprocal relationship between the ability of a cell to retain TMRM and reject a normally cell-impermeable dye is again consistent with our findings, and obviously does not prove that TMRM loss occurs before SP. Furthermore, it does not exclude any order of the two critical events in time.

Perhaps Davidson et al. [24] provided stronger evidence that MPT precedes SP. They used the cell-impermeable dye calcein as an SP indicator and presented a snapshot of a myocyte devoid of both F_TMRM_ and calcein (a “black cell”) stating that after TMRM loss the cell continued rejecting calcein “for minutes” (see Fig 1C in [24]). However, no statistical treatment of this information was provided. Finally, Wang et al. [16] also presented snapshots of confocal images demonstrating “black cells” during reperfusion which lost TMRM but did not uptake the normally cell impermeable Evans blue dye (EBD) (see Fig 2A in [16]). However, no assessment of T_MPT_ and T_SP_ was reported.

In our study, we did not observe any “black cells” in all hearts subjected to ischemia/reperfusion (*Control*, *CsA*, and *No_BDM* groups), that is, all cells devoid of TMRM had a detectable presence of YO-PRO1. The discrepancy between our study and those by Davidson et al. [24] and Wang et al. [16] can be explained by several factors: (1) species (rabbit in our study versus mouse in theirs); (2) type of metabolic stress (80-min ischemia in our study versus 30-min ischemia [16] or 45-min hypoxia [24]); (3) different indicator of sarcolemmal integrity (YO-PRO1 versus EBD [16] or calcein [24]). The advantage of YO-PRO1 over calcein and EBD is twofold: (1) smaller molecular size (the Stokes radius of 0.48 nm vs. 0.6 nm for calcein and 1.3 nm for Evans Blue) and (2) larger contrast between the extracellular and intracellular signal. YO-PRO1 fluorescence increases over 100-fold upon entering cells and binding to nucleic acids, which are present almost exclusively in the intracellular space. Calcein and EBD fluoresce the same way inside and outside cells. These properties of YO-PRO1 may permit a faster detection of SP by this dye as compared to other SP indicators. This, in turn, may affect conclusions regarding the apparent order of MPT and SP.

### Mechanistic interpretation of the outcomes

The prevailing view of ischemia/reperfusion injury posits that the pivotal step in the path to cell death is MPT, which is triggered primarily by a combination of cellular Ca^2+^ overload and increased mitochondrial ROS production. Ca^2+^ overload is commonly attributed to a coupled action of sodium-proton exchanger and sodium-calcium exchanger in response to abrupt restoration of extracellular pH upon reperfusion, whereas a burst of ROS is attributed to rapid oxidation of NADH [30] or succinate [31] by a possibly damaged [32] electron transport chain. Ca^2+^ overload and bursts of mitochondrial ROS are expected to occur within minutes of reperfusion [32]. This has led to the notion that MPT also occurs within minutes of reperfusion. For the last twenty years, the strongest support for this time window of MPT has been a study from the Halestrap group demonstrating that the largest increase in mitochondrial uptake of deoxyglucose, which is normally unable to cross the inner mitochondrial membrane, occurs between 2 and 5 minutes of reperfusion [33]. However, confocal imaging studies, using an abrupt and irreversible loss of F_TMRM_ in discrete myocytes as an indicator of MPT showed that these events spread over a period of at least 20–30 minutes [23, 24]. In the current study, we observed the apparent MPT events between 9 and 131 minutes of reperfusion. At least some of those events cannot be explained by Ca^2+^ overload and/or ROS occurring upon restoration of perfusion and re-introduction of oxygen. In addition, a recent study from the Halestrap group demonstrated that the rise of [Ca^2+^]_cyt_ is not immediate upon reperfusion in beating hearts subjected to 30 min of global ischemia, but occurs at a variable delay ranging between 2 and 20 min in different experiments (see Fig 10A in [34]). To reconcile this finding with their earlier observations [33], the authors speculated that the observed [Ca^2+^]_cyt_ elevations are a consequence rather than the cause of MPT [34]. Another study using confocal microscopy and genetically-encoded [Ca^2+^]_cyt_ indicator revealed slow Ca^2+^ waves occurring in the time range 0–50 min of reoxygenation after 40 min of anoxia in mouse hearts [24]. However, in this study the apparent MPT events were secondary to the local increases in [Ca^2+^]_cyt_. The mechanism of [Ca^2+^]_cyt_ elevations occurring at variable times of reperfusion [24, 34] remains unexplained.

Summarizing this, the timing and triggers of MPT during reperfusion in whole hearts remain controversial. Our results raise a possibility that timing of MPT is determined by a critical level of sarcolemmal permeability. For the first time, we show that the abrupt and irreversible dissipation of ΔΨ_m_ reported by the cellular loss of TMRM is, on average, overlapping with cellular uptake of the normally cell-impermeable dye, YO-PRO1. Uptake of YO-PRO1 indicates the presence of pores with a diameter of at least 1 nm in the sarcolemma. Depending on the sensitivity of the imaging system and the position of the imaging plane with respect to the pore(s) location, we may or may not be able to detect the earliest uptake of YO-PRO1 ahead of MPT. However, even a single pore of this size should permit a large influx of Ca^2+^ ions, possibly allowing local concentrations of Ca^2+^ to increase to levels sufficient to trigger MPT, at least in the mitochondria close to the sarcolemmal pore/defect. After that, the process of MPT may spread along the cell due to the well-established ROS-induced ROS-release mechanism [29]. Indeed, most of the time we observed MPT as wave propagating from one end of the cell (see S7 Fig) and this is consistent with the earlier observations [23]. We do not have convincing evidence that YO-PRO1 uptake also propagated along the cell. One possibility is that SP did occur first at a cell end, but our temporal and spatial resolution was not sufficient to detect this. In many cases the earliest YO-PRO1 uptake was observed in nuclei, simply due to the largest concentration of nucleic acids in this organelle; clearly YO-PRO1 had to cross the sarcolemma at some earlier time, invisible to us. Another possibility is that SP occurs uniformly over the entire surface of the cell, but for some reason mitochondria at a cell end are more vulnerable. In any event, the wave-like pattern of MPT occurring in ventricular myocytes upon reperfusion awaits explanation.

The fact that the MPT pore inhibitor, CsA, postpones the loss of ΔΨ_m_ with respect to the onset of SP (see Fig 6), supports the view that the observed loss of ΔΨ_m_ is due to MPT pore opening. It is important to understand that CsA is a not a blocker, but rather a modulator, of the MPT pore. It reduces sensitivity of the pore to [Ca^2+^] but cannot prevent MPT pore opening when a sufficiently high [Ca^2+^] in the vicinity of the pore is achieved [7]. Assuming that CsA increases the [Ca^2+^] threshold of MPT, in the presence of CsA it would take longer for [Ca^2+^]_cyt_ to reach the threshold of MPT triggering than in the absence of the drug, but eventually MPT could not be preventable if the sarcolemmal pores/defects expose at least some mitochondria to extracellular [Ca^2+^].

Importantly, however, the spread of MPT occurring during natural reperfusion events does not cross cell boundaries. This is consistent with a significant elevation of [Ca^2+^]_cyt_ due to sarcolemmal leak, leading to closure of gap junctional channels and isolating the troubled cell from its neighbors. It can be argued that this process is beneficial for the bulk myocardium because it limits the spread of cell death. In contrast, MPT induced by H_2_O_2_ propagates freely from cell to cell, leading to global loss of ΔΨ_m_ (see Fig 4 and S4 Movie). This principal difference between naturally occurring and induced MPT further reinforces the idea that MPT, per se, is not sufficient to explain the observed pattern of cell death during reperfusion.

Lastly, the experiments using digitonin revealed that when the sarcolemma is permeable enough to pass YO-PRO1, then there is either a short, or no, delay between YO-PRO1 uptake and TMRM loss. Thus, digitonin application largely reproduces the natural events occurring during reperfusion. Even though there was on average a longer time gap between SP and MPT in *Control* group than in *Digi* group, neither gap was significantly different from zero. One possibility is that the size and/or distribution of pores is different between naturally occurring and digitonin-induced SP.

The nature of cellular permeabilization reported by YO-PRO1 remains unclear. In fact, it may indicate either sarcolemmal defects or opening of large pores such as connexin hemichannels, pannexin channels, or purinergic receptor channels (for review, see [35]). Sarcolemmal defects could be due to a number of factors including disruption of the lipid bilayer order by amphiphilic lipids accumulating during ischemia [36] and deficiencies in membrane repair mechanisms mediated via dystrophin-glycoprotein complex, dyspherlin and MG53 [15–17, 37–39]. However, it seems unlikely that the observed SP is secondary to “hypercontracture” [40]. Even though the majority of cells undergoing SP showed some contraction, it was inconsistent between cells and did not amount to the definition of “hypercontracture” describing extreme shortening (> 60% of cell length) observed in isolated cardiomyocytes subjected to simulated ischemia/reperfusion protocols [14]. However, one must take into account that the cell shape in the myocardial syncytium is the net result of all pulling forces applied to this cell and the cell’s mechanical compliance. Even if a cell shape does not appreciably change, we cannot exclude that there is a significant pulling force applied to some parts of the cell. In that regard, it is interesting that the MPT often was first visible at a cell end (see S7 Fig). It is tempting to speculate that the cell-to-cell contacts may be disrupted in such a way that they cause SP, initiating the path to myocyte death.

Even though, in our opinion, the findings of this study suggest a mechanistic vector from SP to MPT, we cannot exclude a different scenario in which there is an upstream event independently causing SP and MPT with a similar delay. The nature of such an alleged event remains highly speculative, however. One interesting possibility is that both events are ultimate results of the execution of necroptotic programing, which, on one hand, can induce the formation of large membrane pores (although not confirmed so far in cardiac myocytes), and on the other hand can promote MPT [41].

### Ischemia duration and the effect of CsA on infarct size

CsA is the most prevalent MPT pore inhibitor in experimental studies and is also the drug used to prevent reperfusion injury in clinical trials. Despite the fact that the majority (but not all [19]) experimental studies reported reduction of infarct size in CsA treated hearts/animals, the clinical trials overall show a lack of benefit in patients undergoing percutaneous coronary intervention (PCI) [21]. One factor responsible for the discrepancy may be the duration of ischemia in experimental studies vs. clinical cases. In the vast majority of experimental studies assessing cardioprotection by CsA, the duration of ischemia was between 30 and 50 minutes. For comparison, in the clinical trials of CsA the estimated average time of ischemia was 4.5 hours [21]. In another study in patients undergoing PCI, an indirect indicator, chest pain duration, was on the average of 3 hours [42]. It was previously shown that the cardioprotective effect of CsA may depend on the duration of ischemia [14]. In that study CsA was protective in isolated rat hearts after 50-min episodes of ischemia, but not after 30-min episodes of ischemia. Abolishment of contraction by BDM reduced infarct size by ~50% in hearts subjected to 30 min of ischemia, and was still protective, but much less so, in hearts subjected to 50 min of ischemia (~20% reduction in infarct size). The authors speculated that in shorter episodes of ischemia hypercontracture is the prevalent mechanism of cell death, whereas in longer episodes MPT is the prevalent mechanism [14]. From this finding, it could be extrapolated that the longer the ischemia duration, the greater the protective effect of CsA is, and the smaller the protective effect of BDM. Our study shows that this is not exactly the case, because after 80-min episodes of ischemia neither CsA nor BDM significantly changed the infarct size as compared to untreated beating hearts. Expectedly, such a long period of ischemia caused a massive infarct, but still, it was not a guaranteed death sentence. The percent of infarct varied greatly between hearts and different ventricular sections (see S6 Fig). The variability of infarct size estimate by TTC staining is rarely exposed in reports, but at least in some cases it may be very large (see for example Fig 3 in [43]: in the control group infarct size fluctuates between 20% to 60%). We cannot exclude that the variability of infarct size increases with the duration of the ischemic episode. Knowing molecular differences between myocytes which survive or die in the same heart after severe ischemia could be extremely useful for understanding fundamental mechanisms of I/R injury (or cell resilience), but it remains difficult to design a study addressing this question.

It is also extremely rarely reported how the color threshold for determination of infarct size in TTC-stained sections is chosen. Most recent reports just state that they quantified the white area as infarct, and the area which was red as undamaged myocardium. This generally means that the distinction between “red” and “white” is done manually by eye unless otherwise stated. In our experience, the TTC staining color is far from being either solely red or white but includes a fair share of pink in between (see Fig 3). Without choosing a fixed threshold for color separation, the assessment of the amount of red or white is quite subjective. In order to alleviate this problem, we selected thresholds separating 3 levels: white (severely infarcted), pink (intermediate) and red (viable). While the color thresholds were selected subjectively based on observations of a few hearts, importantly, the chosen thresholds were then applied the same way to all hearts. We found that for all 3 levels of damage, the variance within each group was larger than between groups, therefore the ANOVA test was not significant. We tried two other sets of cut-off levels in the color distribution histogram, but it did not change the outcome (not shown).

It remains unknown whether the shades of pink from TTC staining reflect different degrees of cell damage. It could also reflect the different amount of time elapsed since the irreversible step, whether it is MPT or SP. These questions, perhaps, deserve further analysis, given the weight assigned to the results of TTC staining in the studies of cardioprotection.

### Clinical implications

The first clinical trial of CsA involving a limited number of patients showed reduction of infarct size when CsA was administered prior to PCI [44], but subsequent larger studies showed the lack of benefit [21]. In a recent extensive review of candidate drugs targeting reperfusion injury in patients it was noted that the much longer duration of ischemia in patients (hours) as compared to experimental studies (< 1 hour) may explain the discrepancy between experimental and clinical outcomes [45]. Our findings confirm this notion, showing a failure of CsA to reduce infarct size after 80 minutes of ischemia in a species (rabbit) in which CsA was protective after 30 min of ischemia [46]. A prior experimental study showed that there is also a lower bound of ischemic time when CsA is protective. Specifically, CsA reduced infarct size after 50 min of ischemia, but not after 30 min of ischemia, in a rat model of ischemia/reperfusion [14]. So, it is possible that CsA will show clinical benefit if administered in a selected cohort of patients with confirmed ischemic times between 30 and 60 minutes. Considering longer, and more common, ischemic episodes, our study suggests that targeting the MPT pore may be ineffective because the critical mechanism of cell death is related to SP, occurring prior to MPT. When the degree of sarcolemmal permeabilization is sufficient to expose at least some mitochondria to [Ca^2+^] in high micromolar range, MPT is probably unavoidable. We believe that future experimental studies should focus on the dynamics of SP after prolonged episodes of ischemia, perhaps using the best techniques to increase the temporal and spatial resolution of SP detection, thus providing important clues as to the possible molecular mechanism of this critical event.

### Study limitations

Our study shares limitations with other studies using confocal imaging in isolated hearts in that the measurements were limited to the superficial epicardial myocytes and were performed in non-beating hearts. It remains possible that the mechanism of cell death is different between epicardium and other layers of ventricular wall. Regarding using heart immobilizers, we did not find a significant difference in the infarct size between beating and BDM-treated hearts, suggesting that BDM is not protective after 80-min episodes of ischemia, even though it was shown to be protective in ischemic episodes lasting up to 1 hour [28]. The temporal resolution of confocal imaging was limited to between 15–60 seconds. Having higher temporal resolution could help to ascertain the order of MPT and SP but was not possible because of overt TMRM bleaching and/or phototoxicity observed at higher frame rates. Cell retention of TMRM is a function of both ΔΨ_m_ and ΔΨ_s_ (potential across the sarcolemma), therefore the interpretation of F_TMRM_ as a ΔΨ_m_ readout should be taken with caution. However, the fact that we (and others [23]) observed loss of TMRM propagating along a cell (see S7 Fig) strongly suggests that it reflected spreading dissipation of ΔΨ_m_, because spread of ΔΨ_s_ over a myocyte is instantaneous at the temporal resolution of our recordings. Moreover, the fact that in the wake of propagating ΔΨ_m_ depolarization the residual F_TMRM_ was indistinguishable from background (S7 Fig), suggests that the ΔΨ_m_ loss in naturally occurring cell transitions was virtually simultaneous with ΔΨ_s_ loss, providing additional evidence for the tight coupling between MPT and SP in injured myocytes.

## Supporting information

S1 Text Legends for all supplemental figures and movies. (DOC)

S1 Fig Detailed description of TTC staining procedure and image analysis. (TIF)

S2 Fig Example of F_TMRM_ loss caused by excessive laser exposure. (TIF)

S3 Fig Example of global F_TMRM_ and F_YO-PRO1_ dynamics throughout the course of I/R. (TIF)

S4 Fig The overlapping dynamics of F_TMRM_ loss and F_YO-PRO1_ gain during reperfusion in 13 myocytes from the control group. (TIF)

S5 Fig Example of consecutive snapshot images of F_TMRM_ and F_YO-PRO1_ from a beating heart. (TIF)

S6 Fig Extended analysis of the TTC staining data used to determine infarct size. (TIF)

S7 Fig Example of propagating wave of F_TMRM_ loss starting at one end of the cell. (TIF)

S8 Fig Effect of protonophore FCCP on F_TMRM_ and F_YO-PRO1_. (TIF)

S1 Movie Depolarization of ΔΨm during no-flow ischemia. (AVI)

S2 Movie Example of an MPT/SP event in a single ventricular myocyte during reperfusion. (AVI)

S3 Movie Example of an observed MPT/SP event during reperfusion in the presence of CsA. (AVI)

S4 Movie H_2_O_2_-induced wave of mitochondrial depolarization. (AVI)

S5 Movie The second example of an observed MPT/SP event during reperfusion in *Control* group. (AVI)

S1 Dataset Cell-averaged F_TMRM_ and F_YO-PRO1_ for cells from *Control* group. (XLSX)

S2 Dataset Cell-averaged F_TMRM_ and F_YO-PRO1_ for cells from *CsA* group. (XLSX)

S3 Dataset Cell-averaged F_TMRM_ and F_YO-PRO1_ for cells from *Digi* group. (XLSX)
