# Supplementary material for: Cyclosporine-insensitive mode of cell death after prolonged myocardial ischemia: Evidence for sarcolemmal permeabilization as the pivotal step
Source: PLoS One. 2018 Jul 5;13(7):e0200301. doi: 10.1371/journal.pone.0200301 (PMC6033462; doi:10.1371/journal.pone.0200301)
Supplement: S1 Text — (DOC) [file pone.0200301.s001.doc]

**Text S1: Legends for all supplemental figures and movies.**

**Supplemental Figures.**

**Figure S1**

**Detailed visual description of TTC staining procedure and image analysis.**

Directly following 3 hours of reperfusion, hearts were axially sliced (approximately 0.5 cm thick) from base to apex. Slices were immediately immersed in 1% weight/volume TTC in PBS and incubated at 37°C for 10 mins. After incubation, slices were placed on a HP Scanjet 5550c scanner and high-resolution images (2400dpi) were obtained for both the apical and basal sides. Each slice was then isolated from the full image to form separate color images, which were then split into the monochrome RGB components using ImageJ software. The green channel component has been shown to provide the highest contrast between viable and infarcted tissue regions compared to the red and blue components [1], and thus was used for the remainder of analysis. The LV was manually segmented and all other pixels were masked out. Using a custom-built MATLAB program, a histogram of the green channel LV was generated and threshold regions below the intensity values of 90, between 90 and 125, and above 125 were computed. These threshold regions correspond with viable, intermediate, and severely infarcted tissue respectively (shown in red, pink, and white). The total number of pixels in each thresholded region were summed, and then divided by the number of pixels comprising the entire LV to determine the overall percentage of each region within its individual slice.

**Figure S2**

**Example of F_TMRM_ loss caused by excessive laser exposure.**

**A** to **C**, merged F_TMRM_/F_YO-PRO1_ images obtained using the 20x lens at consecutive time points as indicated. Note the gradual but significant decrease in F_TMRM_ between 40 and 60 min of reperfusion (possibly due to bleaching, but true ΔΨ_m_ loss cannot be excluded), without F_YO-PRO1_ increase. **D**, a switch from the 20x to 10x lens reveals that the loss of F_TMRM_ is aligned with the boundaries of the field of view taken with the 20x lens (white brackets) and is therefore artifactual.

**Figure S3**

**Example of global F_TMRM_ and F_YO-PRO1_ dynamics throughout the course of I/R.**

A bird’s eye view of changes in ΔΨ_m_ and cellular permeability in the field of view (450 x 450 μm, ~80-100 cells) throughout the entire course of ischemia/reperfusion. Green, F_TMRM_; orange, F_YO-PRO1_. We applied spatial Fourier transform to F_TMRM_ images and tracked changes in the spectral peak corresponding to periodic packaging of interfibrillar mitochondria with the period of ~2 μm [2]. F_YO-PRO1_ was not analyzed, but it can be seen that Y_O-PRO1_ uptake occurred only during reperfusion and only in some cells. Panels **A** to **F** show the merged F_TMRM_/ F_YO-PRO1_ images (left) and the respective spectral profiles derived from F_TMRM_ images (right) for selected time points indicated in **G**. **G**, the time course of the Mitochondrial Peak Area (MPA) computed from the spectral profiles (green). The discontinuities in the curve indicate changes in the field of view. The same field of view was tracked through the ischemic episode (time points **A**-**D**), but was lost upon reperfusion due to massive changes in the heart shape. Three different fields of view were imaged during reperfusion. The vertical dashed line indicates the time of 50% decrease of MPA which was used as the estimate of time of ischemic ΔΨ_m_ depolarization. The typical sequence of events represented here included ΔΨ_m_ depolarization between 20 and 40 min of ischemia and a significant recovery of ΔΨ_m_ upon reperfusion. However, during reperfusion some cells completely lost TMRM and became permeable to YO-PRO1, suggesting the occurrence of both MPT and SP in these cells.

**Figure S4.**

**The overlapping dynamics of F_TMRM_ loss and F_YO-PRO1_ gain during reperfusion in 13 myocytes from the *Control* group.**

The cells are in arbitrary order to optimize display. Green and orange curves represent cell-averaged F_TMRM_ and F_YO-PRO1_, respectively, in absolute levels of fluorescence intensity yielded by the confocal system. The lowest values on the y-axes represent the lowest level of the respective signal in the field of view, always found in the extracellular space. Vertical green and orange dashed lines show the time of detected MPT and SP, respectively, according to the detection criteria detailed in the methods. The time is shown in minutes with respect to the onset of reperfusion. Despite variability of the dynamics among different myocytes, note that in 12/13 cells (cell #5 is an outlier according to Grubbs test) there is a clear increase in the level of F_YO-PRO1_ by the time of complete F_TMRM_ loss, suggesting that the two critical processes are overlapping and cannot be separated in time. See text for detail.

**Figure S5.**

**Example of consecutive snapshot images of F_TMRM_ and F_YO-PRO1_ from a beating heart**

Snapshot images obtained using the fast (video rate) scan mode (**A**-**C**). Green, fluorescence of TMRM (F_TMRM_); orange, fluorescence of YO-PRO1 (F_YO-PRO1_). Note that even though fast scan mode afforded sharp images in contracting hearts, the field of view is ever shifting (see changing positions of the individual myocyte outlined with white), decreasing the chance of continuously following any single cell for long periods of time. Note, however, that still images do not show “black cells” (those devoid of both TMRM and YO-PRO1) suggesting that there is no time gap between TMRM loss and YO-PRO1 uptake.

**Figure S6.**

**Extended analysis of the TTC staining data used to determine infarct size.**

**A**, Percent of severely infarcted area (white) obtained from all sections in all available hearts from groups *No_BDM*, *Control*, and *CsA*. Each column of points represents data from a single heart. Note a large variability of infarct percent between hearts as well as between sections of the same heart. **B**, despite overall variability shown in **A**, there was a significant difference in percent of viable tissue (red) between the most apical (squares) and most basal (circles) sections in each heart in every analyzed group. See also Figure 3 in the main text.

**Figure S7.**

**Example of a propagating wave of F_TMRM_ loss starting at one end of the cell**

The data shown is from a single cell (Cell #13 from *Control* group, Figure S4 and Movie S5) outlined in white in panel **G**. **A-F**, consecutive snapshots of F_TMRM_ at 1-minute intervals. Note a fairly constant speed of wave propagation, ~20 μm/min. The wave did not propagate to neighboring cells. This pattern was observed in the majority of cells from both *Control* and *CsA* groups. The fact that the wave propagates so slowly, and the fact that in the wake of the wave the level of F_TMRM_ is indistinguishable from background, strongly and simultaneously suggest that (1) the wave reflects ΔΨ_m_ loss, because the cell membrane potential cannot propagate so slowly, and (2) that during this process the sarcolemmal potential is very low or nonexistent. These inferences further support the assumption that MPT is virtually simultaneous with SP (which would be the easiest way to explain the presumable loss of sarcolemmal potential).

**Figure S8.**

**Effect of protonophore FCCP on F_TMRM_ and F_YO-PRO1_**

The time is given in minutes since the onset of observation beginning at 33 min of reperfusion. Green, fluorescence of TMRM (F_TMRM_); orange, fluorescence of YO-PRO1 (F_YO-PRO1_). The lowest values on the y-axes represent the lowest level of the respective signals in the field of view, always found in the extracellular space. The orange horizontal dashed line is the average of maximal cellular F_YO-PRO1_ levels observed during naturally occurring MPT/SP transitions (see Figure S4). It appears that the lowest concentration (1 μM, arrow) was sufficient to trigger a slow exponential loss of F_TMRM_, but none of the concentrations used caused any YO-PRO1 uptake. Thus, a forced ΔΨ_m_ depolarization during was not sufficient to cause SP.

**Supplemental Movie Legends**

**Movie S1**

**Depolarization of ΔΨm during no-flow ischemia.**

Merged images (green, F_TMRM_; orange, F_YO-PRO1_) were acquired every 30 seconds during ischemia, beginning at min 47 in Figure S3, G (27 min ischemia) for a total duration of 15 min. The image sequence is accelerated to 4 frames per second for visual purposes. Notice the grid-like pattern of F_TMRM_ suggesting polarized mitochondria at the start of the movie. The first noticeable event is contracture of cells creating a “wavy” pattern especially visible in the center of the view. Subsequently, there is a transition from rough to smooth appearance of the TMRM image indicating dissipation of ΔΨ_m_ which is simultaneous with straightening out and relaxation of myocytes. Cells remain in this state, maintaining membrane integrity, until the end of the ischemic episode and do not become permeable to YO-PRO1.

**Movie S2**

**Example of an MPT/SP event in a single ventricular myocyte during reperfusion.**

This event is in the same cell as shown in Figure 1 and Figure S4 (Cell #9 from *Control* group). Snapshots in this movie were obtained every minute, beginning at time point ***a*** of Figure 1 (16 min reperfusion), and lasting throughout the rest of the tracking period (47 min reperfusion). F_TMRM_ and F_YO-PRO1_ (green and orange, respectively) are shown merged together with an increased frame rate of 3 frames per second. Yellow arrows at the start of the movie indicate the cell of interest. Note the virtual simultaneity of a rapid loss of F_TMRM_ propagating from one cell end to the other (right to left) and uptake of F_YO-PRO1_.

**Movie S3**

**Example of an observed MPT/SP event during reperfusion in the presence of CsA**.

This event is the same cell shown in Figure 2, starting at 19.5 mins of reperfusion and continuing through 33 mins. Snapshots are obtained every 30 secs, the F_TMRM_ and F_YO-PRO1_ (green and orange, respectively) channels are merged, and the frame rate is increased to 3 frames per sec for visual purposes as in Movie S2. White arrows at the beginning denote the cell of interest. Note the visible increase in F_YO-PRO1_ while F_TMRM_ remains bright for several mins before finally declining. This again suggests that SP is the primary event, and that CsA postpones the MPT event compared to transitions in the control group, but cannot prevent it.

**Movie S4**

**H_2_O_2_-induced wave of mitochondrial depolarization**

The same but slightly wider area as in Figure 4 (green, F_TMRM_; orange, F_YO-PRO1_). The movie begins 6 min after the end of 200 μM H_2_O_2_ application. Images were captured every 30 seconds and sped up to 4 frames per second. The observed wave of MPT crosses cell boundaries and propagates as a front, leaving cells in its wake which maintain their sarcolemmal potential and do not immediately undergo SP. Thus, MPT induced by H_2_O_2_ does not replicate natural MPT/SP events. This movie also demonstrates the tissue movement and plane changes that occur with H_2_O_2_-triggered MPT, making it difficult to track the same areas of tissue for extended periods of time.

**Movie S5**

**The second example of an observed MPT/SP event during reperfusion in *Control* group**.

The cell of interest is the same as cell #13 in Figure S4 and also Figure S7, and is indicated by white arrows at the beginning of the movie (116 min reperfusion). Images (green, F_TMRM_; orange, F_YO-PRO1_) were acquired every 1 min for 50 min total, and the frame rate was increased to 3 frames per second for visual purposes. Notice again that the cell loses F_TMRM_ in a wave-like pattern from one cell end to the other (left to right), while simultaneously undergoing SP evidenced by increasing F_YO-PRO1_. Importantly, the uptake of YO-PRO1 becomes visible in the left-hand end of the cell while the right-hand end of the cell still maintains polarized mitochondria, highlighting the spatiotemporal overlap of MPT and SP in the same cell.

**References**

1. Goldlust EJ, Paczynski RP, He YY, Hsu CY, Goldberg MP. Automated Measurement of Infarct Size With Scanned Images of Triphenyltetrazolium Chloride–Stained Rat Brains. Stroke. 1996;27(9):1657-62. doi: 10.1161/01.str.27.9.1657.

2. Venable PW, Taylor TG, Sciuto KJ, Zhao J, Shibayama J, Warren M, et al. Detection of mitochondrial depolarization/recovery during ischaemia--reperfusion using spectral properties of confocally recorded TMRM fluorescence. J Physiol. 2013;591(Pt 11):2781-94. doi: 10.1113/jphysiol.2012.248153. PubMed PMID: 23529126; PubMed Central PMCID: PMC3690686.
