# Supplementary figures and images for: Cyclosporine-insensitive mode of cell death after prolonged myocardial ischemia: Evidence for sarcolemmal permeabilization as the pivotal step
Source: PLoS One. 2018 Jul 5;13(7):e0200301. doi: 10.1371/journal.pone.0200301 (PMC6033462; doi:10.1371/journal.pone.0200301)

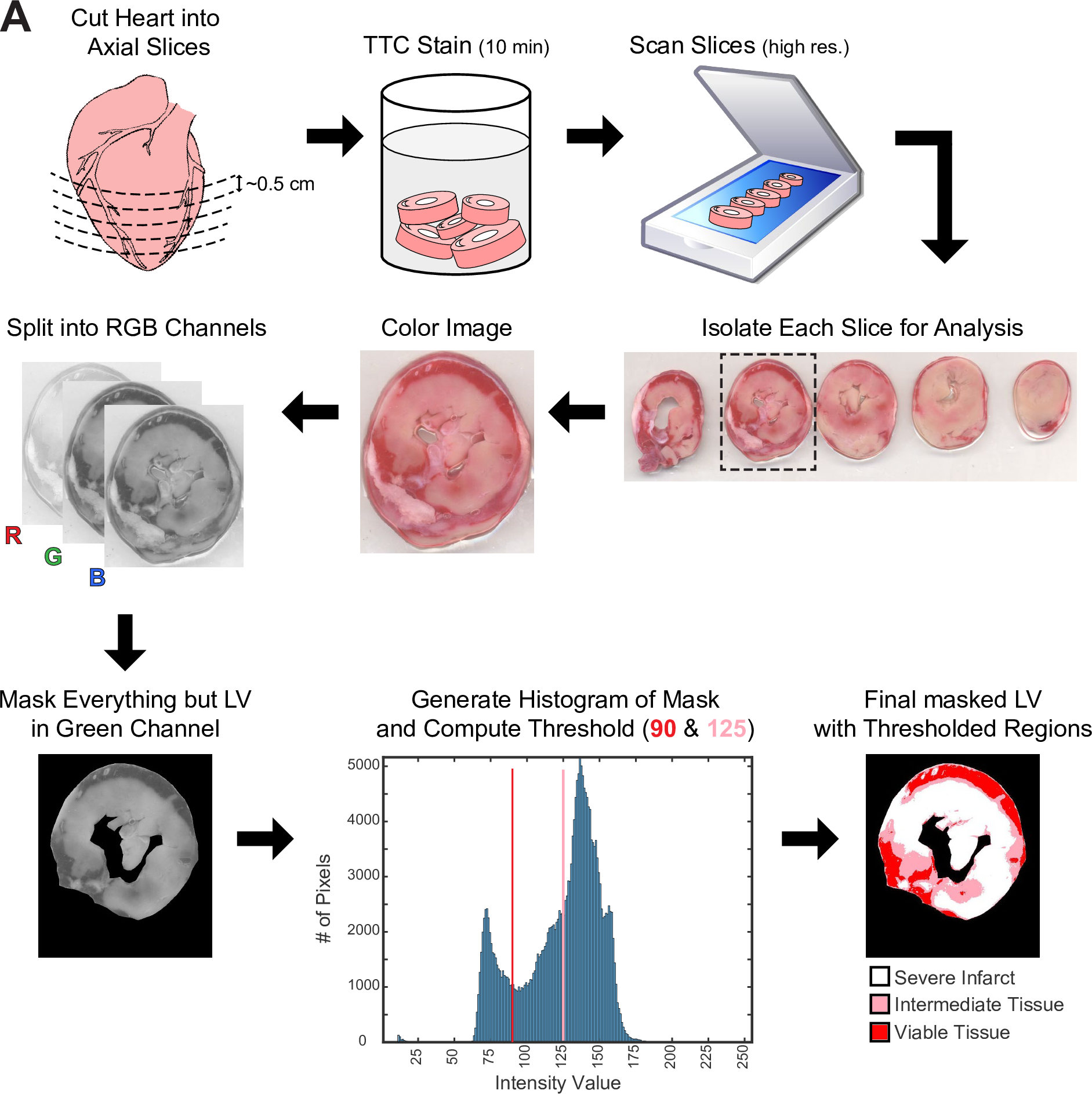

Supplement: S1 Fig — (TIF) [file pone.0200301.s002.tif]

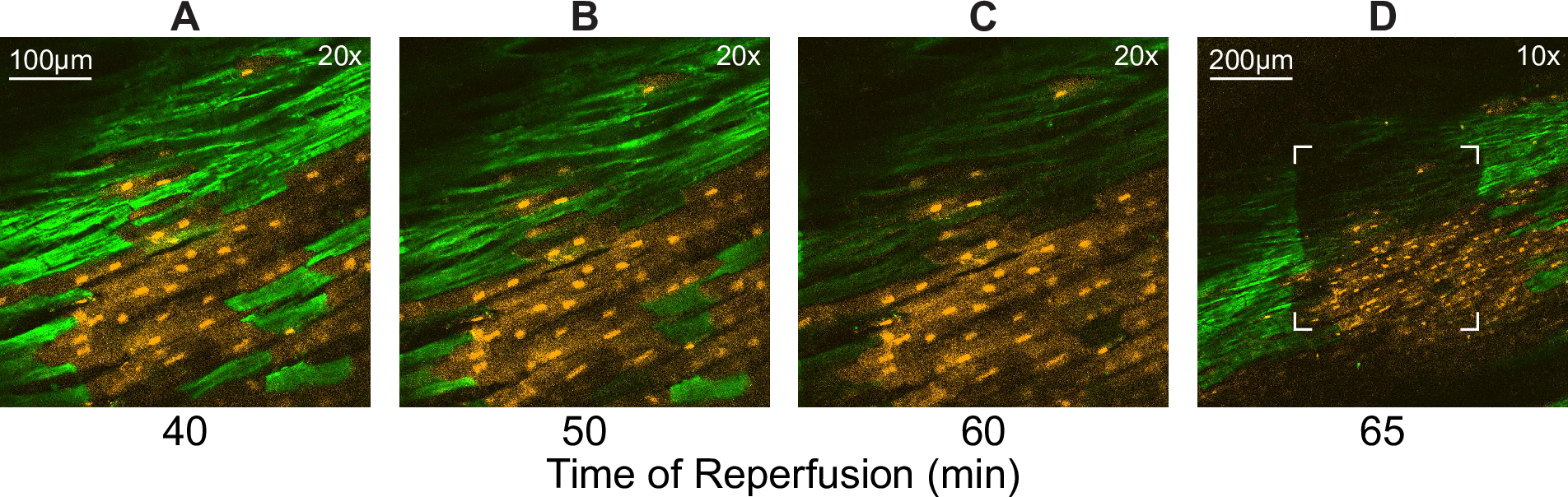

Supplement: S2 Fig — (TIF) [file pone.0200301.s003.tif]

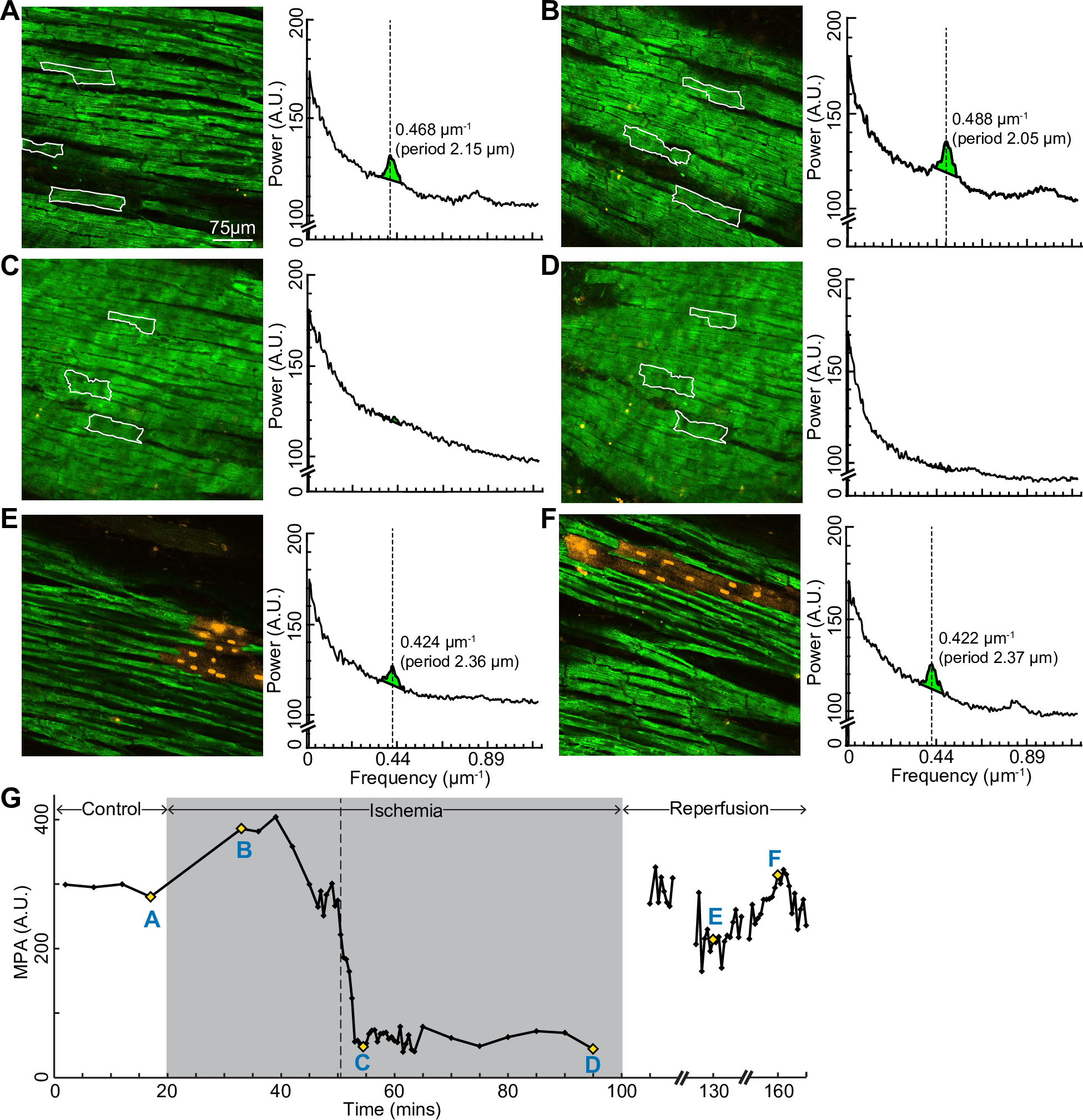

Supplement: S3 Fig — (TIF) [file pone.0200301.s004.tif]

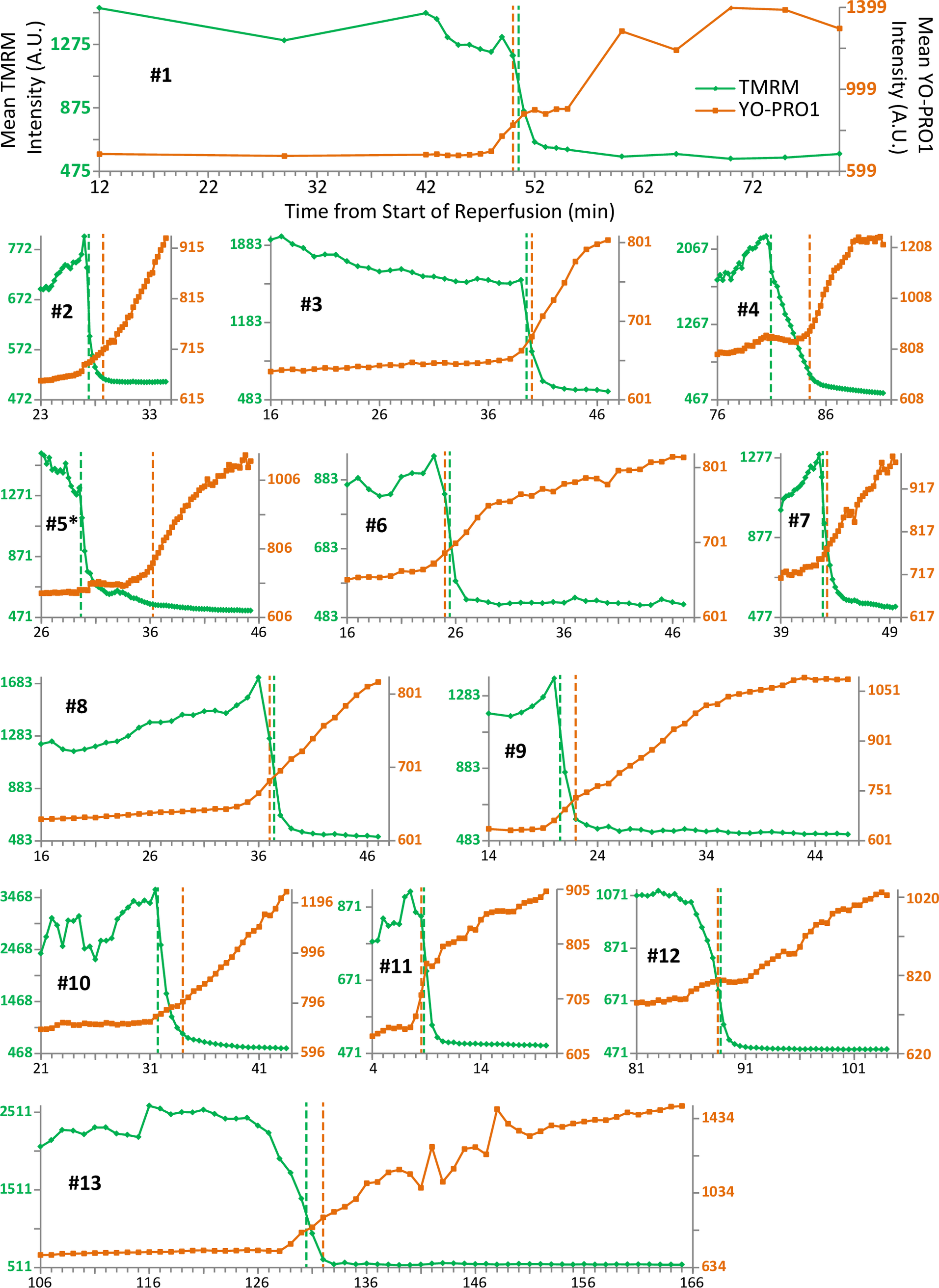

Supplement: S4 Fig — (TIF) [file pone.0200301.s005.tif]

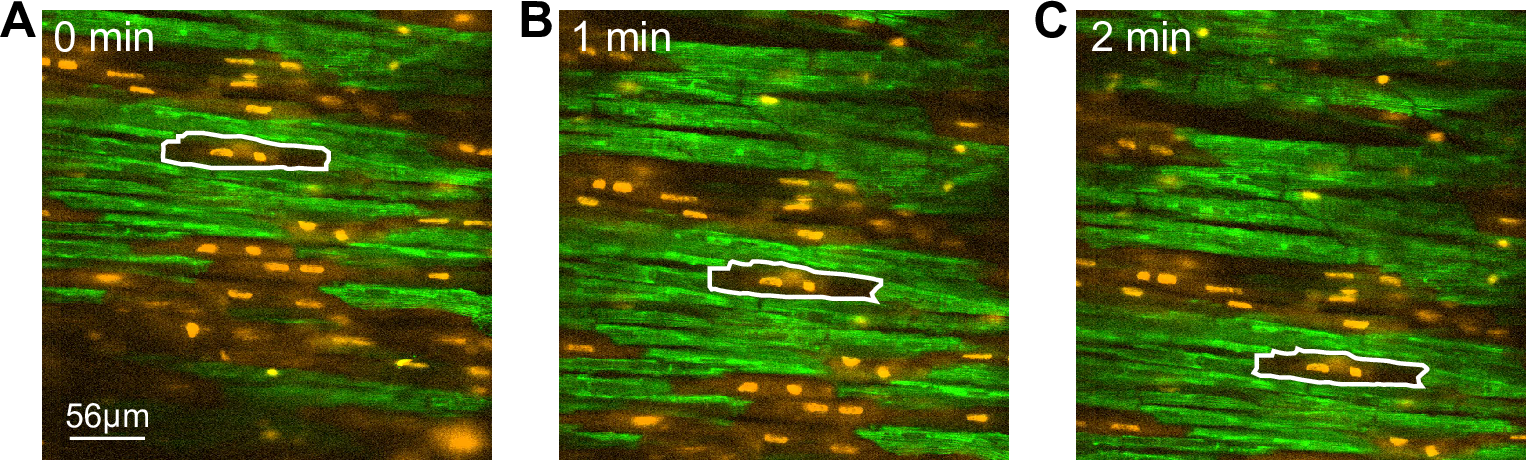

Supplement: S5 Fig — (TIF) [file pone.0200301.s006.tif]

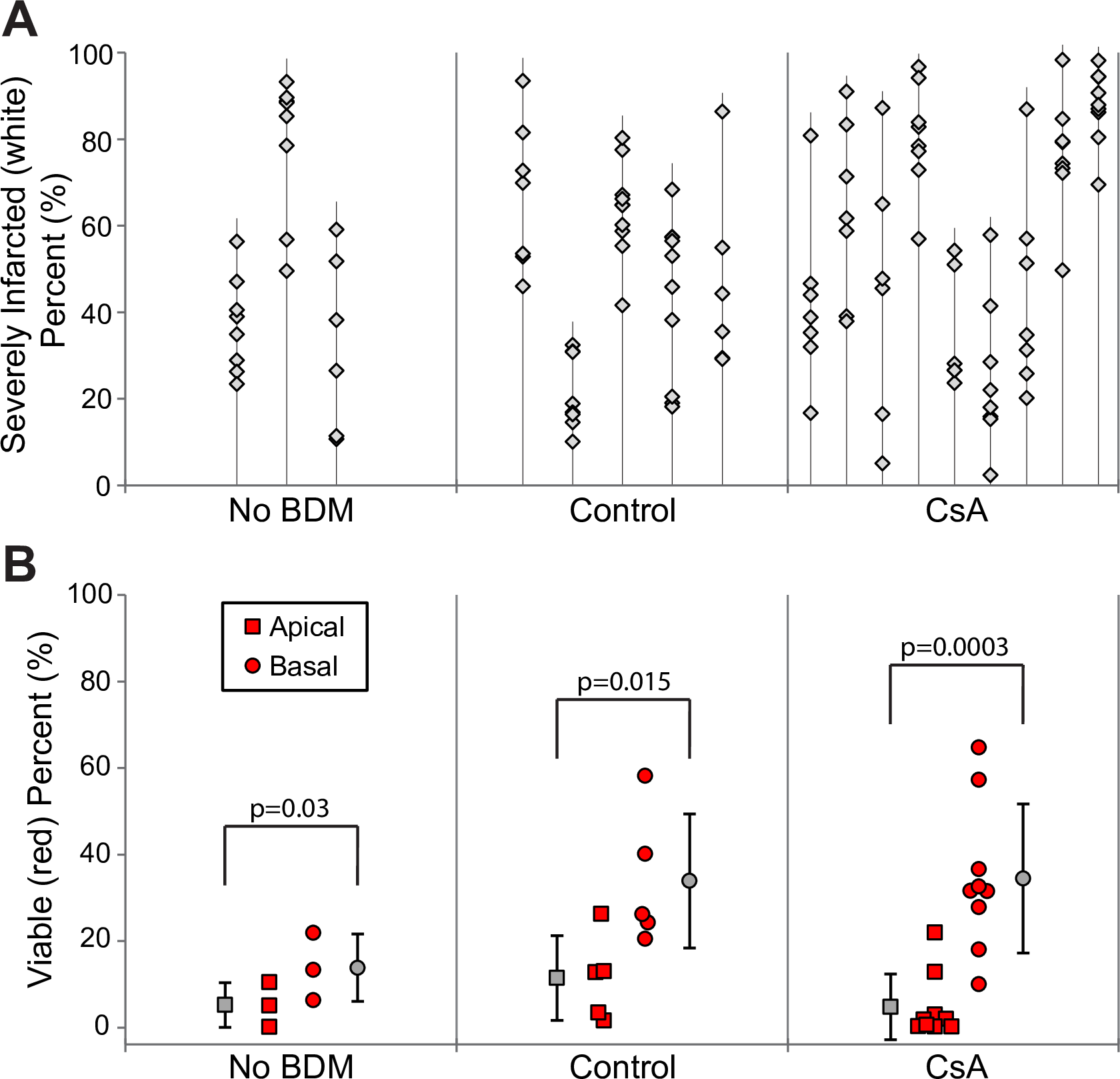

Supplement: S6 Fig — (TIF) [file pone.0200301.s007.tif]

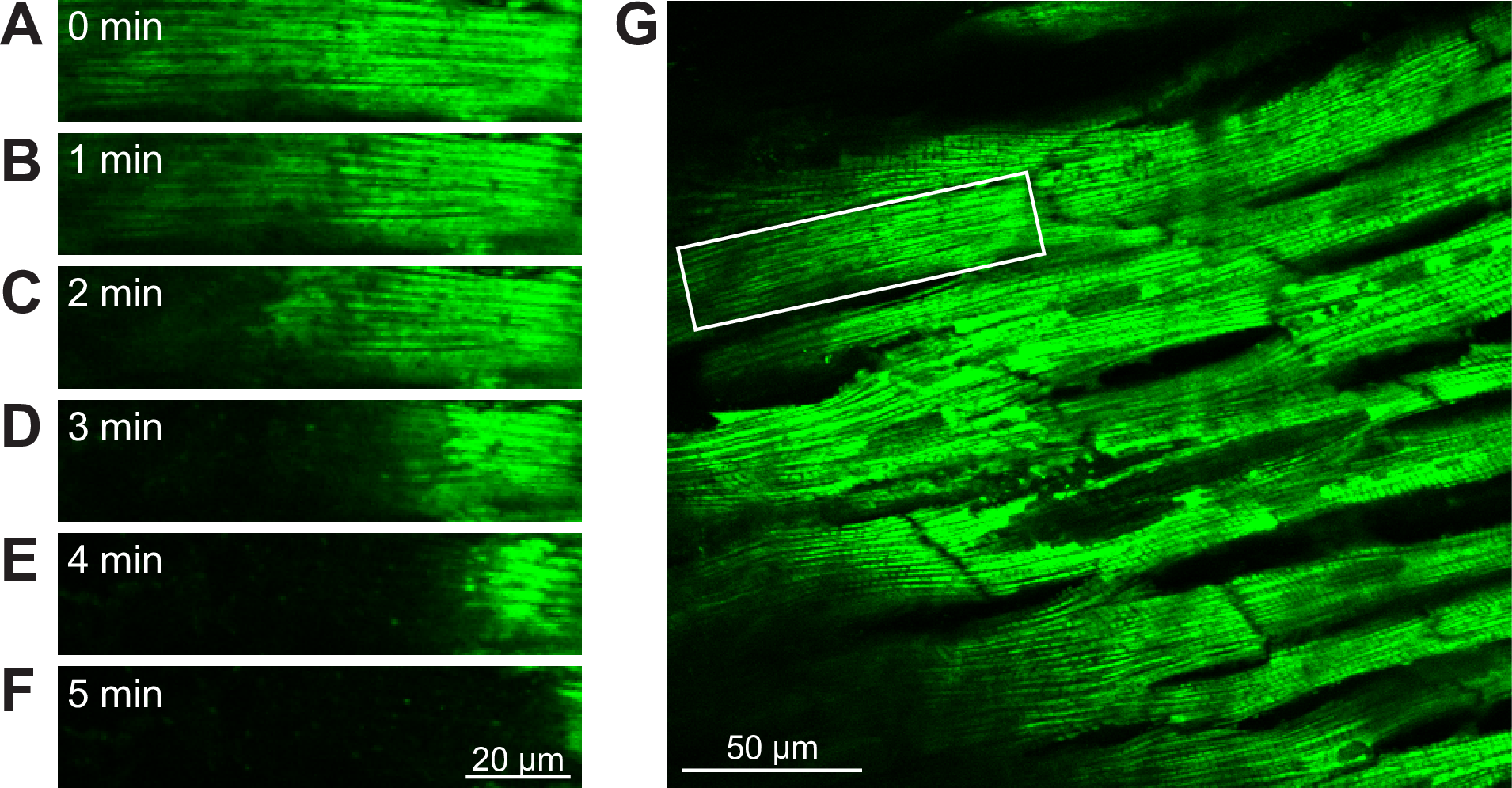

Supplement: S7 Fig — (TIF) [file pone.0200301.s008.tif]

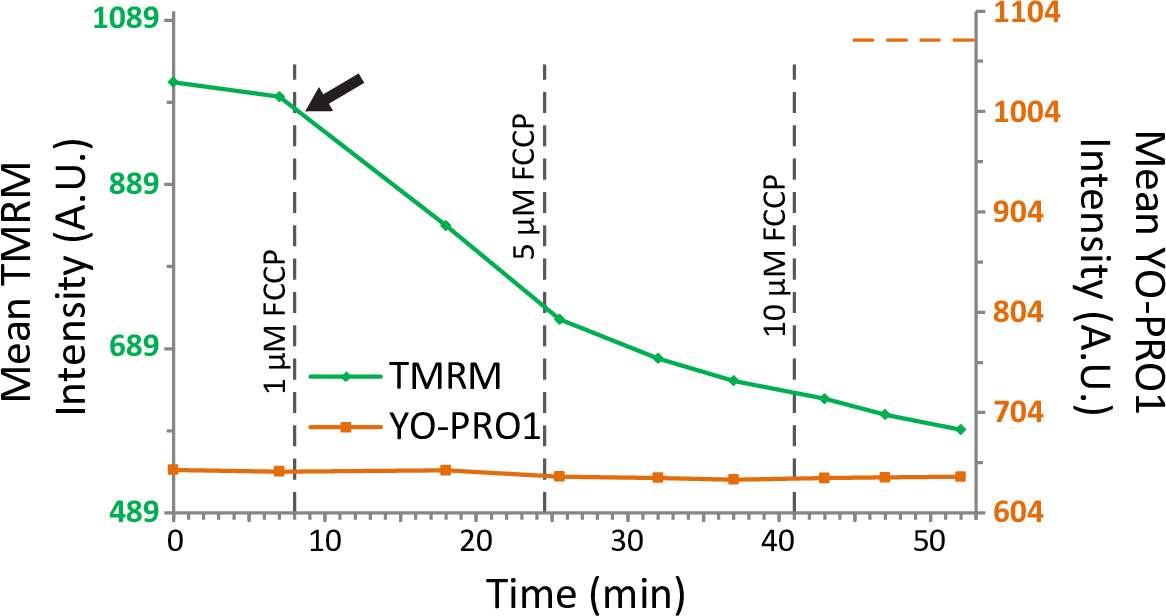

Supplement: S8 Fig — (TIF) [file pone.0200301.s009.tif]
